# Design and implementation of a scalable high-performance computing (HPC) cluster for omics data analysis: achievements, challenges and recommendations in LMICs

**DOI:** 10.1093/gigascience/giae060

**Published:** 2024-08-22

**Authors:** Kais Ghedira, Oussema Khamessi, Chaima Hkimi, Selim Kamoun, Nader Dhamer, Kamel Daassi, Wassim Ben Salah, Houcemeddine Othman, Wahbi Belhadj, Youssef Ghorbal

**Affiliations:** Laboratory of Bioinformatics, Biomathematics and Biostatistics, LR16IPT09, Institut Pasteur de Tunis, University of Tunis El Manar, Tunis 1002, Tunisia; Laboratory of Bioinformatics, Biomathematics and Biostatistics, LR16IPT09, Institut Pasteur de Tunis, University of Tunis El Manar, Tunis 1002, Tunisia; High Institute of Biotechnology of Sidi Thabet, University of Manouba, Ariana BP-66, Manouba 2010, Tunisia; Laboratory of Bioinformatics, Biomathematics and Biostatistics, LR16IPT09, Institut Pasteur de Tunis, University of Tunis El Manar, Tunis 1002, Tunisia; High Institute of Biotechnology of Sidi Thabet, University of Manouba, Ariana BP-66, Manouba 2010, Tunisia; Laboratory of Bioinformatics, Biomathematics and Biostatistics, LR16IPT09, Institut Pasteur de Tunis, University of Tunis El Manar, Tunis 1002, Tunisia; High Institute of Biotechnology of Sidi Thabet, University of Manouba, Ariana BP-66, Manouba 2010, Tunisia; Laboratory of Bioinformatics, Biomathematics and Biostatistics, LR16IPT09, Institut Pasteur de Tunis, University of Tunis El Manar, Tunis 1002, Tunisia; Direction Informatique de l’Institut Pasteur de Tunis, Institut Pasteur de Tunis, University of Tunis El Manar, Tunis 1002, Tunisia; Direction Informatique de l’Institut Pasteur de Tunis, Institut Pasteur de Tunis, University of Tunis El Manar, Tunis 1002, Tunisia; Direction Informatique de l’Institut Pasteur de Tunis, Institut Pasteur de Tunis, University of Tunis El Manar, Tunis 1002, Tunisia; Sydney Brenner Institute for Molecular Bioscience, Faculty of Health Sciences, University of the Witwatersrand, Johannesburg 2193, South Africa; The European Bioinformatics Institute–European Bioinformatics Institute (EMBL-EBI), Hinxton CB10 1SD, United Kingdom; HPC Core Facility of the Institut Pasteur Paris, 25-28 Rue du Dr Roux, 75015 Paris, France

**Keywords:** bioinformatics, HPC, Tunisia, infrastructure, computational power, computing cluster

## Abstract

**Background:**

The advent of high-throughput technologies, including cutting-edge sequencing devices, has revolutionized biomedical data generation and processing. Nevertheless, big data applications require novel hardware and software for parallel computing and management to handle the ever-growing data size and analysis complexity. On-premise, high-performance computing (HPC) is increasingly used in biomedical research for big data stewardship.

**Findings:**

In this work, we present Tunisia’s first high-performance computational infrastructure for omics research.

**Method:**

We highlight measurements and recommendations that may help institutions in other low- and middle-income countries that are eager to implement local HPC in facilities for bioinformatics research and omics data analyses.

Key Points:

- Successful implementation of high-performance computing (HPC) within low- and middle-income countries (LMICs), showcasing its vital role in omics research.
- Addressing challenges and barriers faced during the HPC implementation process in the context of LMICs.
- Effective recommendations for HPC infrastructure implementation for omics sciences within LMICs.

## Background

Over the past 2 decades, advancements in high-throughput technologies have resulted in a massive accumulation of omics data, driving biomedical sciences into a big data era [1, 2]. Consequently, robust high-performance computing (HPC) infrastructure has become mandatory to efficiently manage and analyze this huge amount of data and to integrate cutting-edge omics and artificial intelligence techniques [3, 4]. In this context, it is vital to acknowledge the disparities faced by researchers in low- and middle-income countries (LMICs) regarding the access and use of such sophisticated computational resources. Open science infrastructures can promote parity among researchers from both poor and developed nations by facilitating the fair and reciprocal exchange of scientific inputs and outputs, emphasizing the need for infrastructures offering computational and data manipulation services [5]. Previous studies have demonstrated the utility of grid and HPC infrastructures for integrative biomedical research [6, 7]. This is indeed the case for our institute, where a number of bioinformatics analyses, including exome analysis, RNA clustering, and RNA sequencing (RNA-seq) data analysis, were performed thanks to this infrastructure [8, 9]. Here, we discuss the implementation of an HPC infrastructure at the Institut Pasteur de Tunis (IPT), aiming to enhance omics data management and analysis.

## Infrastructure Implementation and Timeline

**Figure 1:**
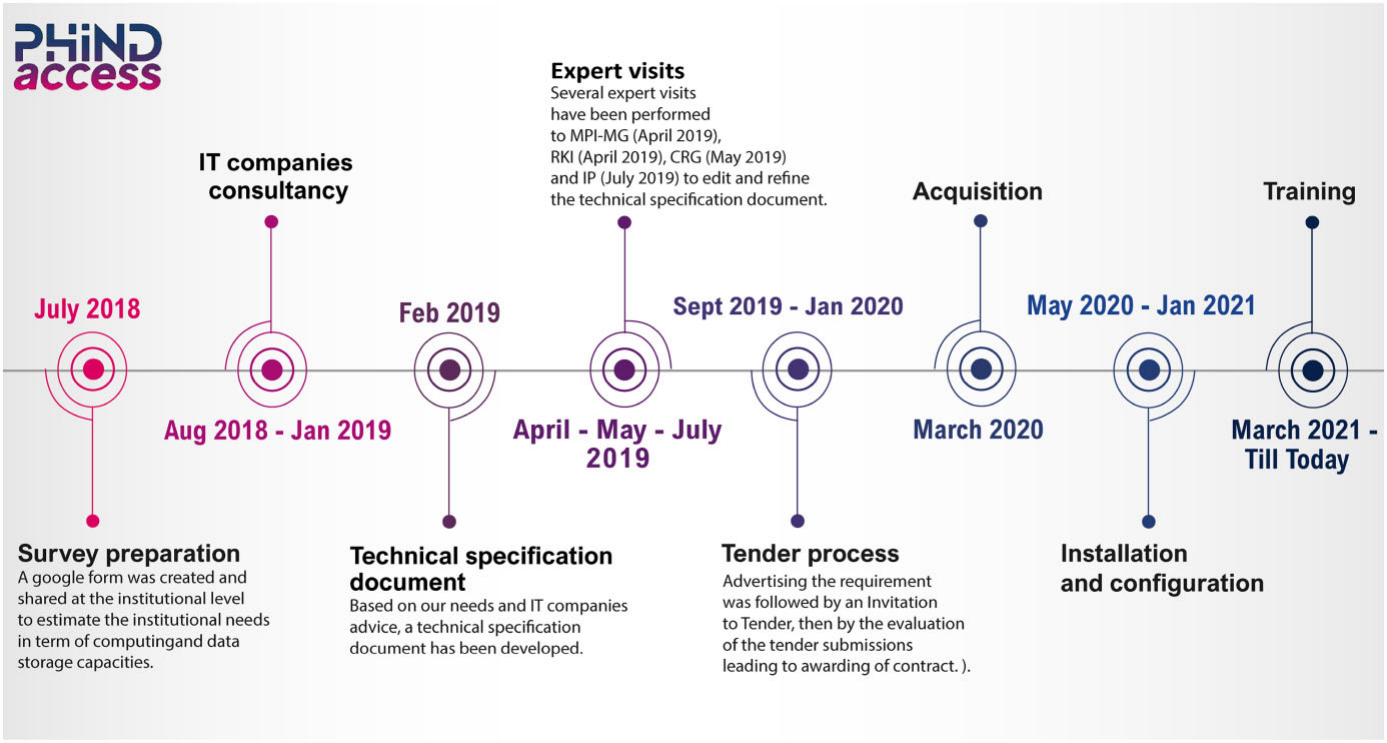
Steps and timeline followed for the acquisition of the infrastructure.

In March 2020, IPT acquired hardware in the frame of the twinning H2020 PHINDaccess project, thanks to the support and funding from the Tunisian Ministry of Scientific Research and Higher Education (MESRS). By January 2021, we introduced “*omics*” at the HPC facility, envisioning it as a cornerstone institutional bioinformatics platform. This initiative aimed to significantly enhance IPT users’ proficiency in omics data analysis and management (Fig. 1).

### HPC configuration

**Figure 2:**
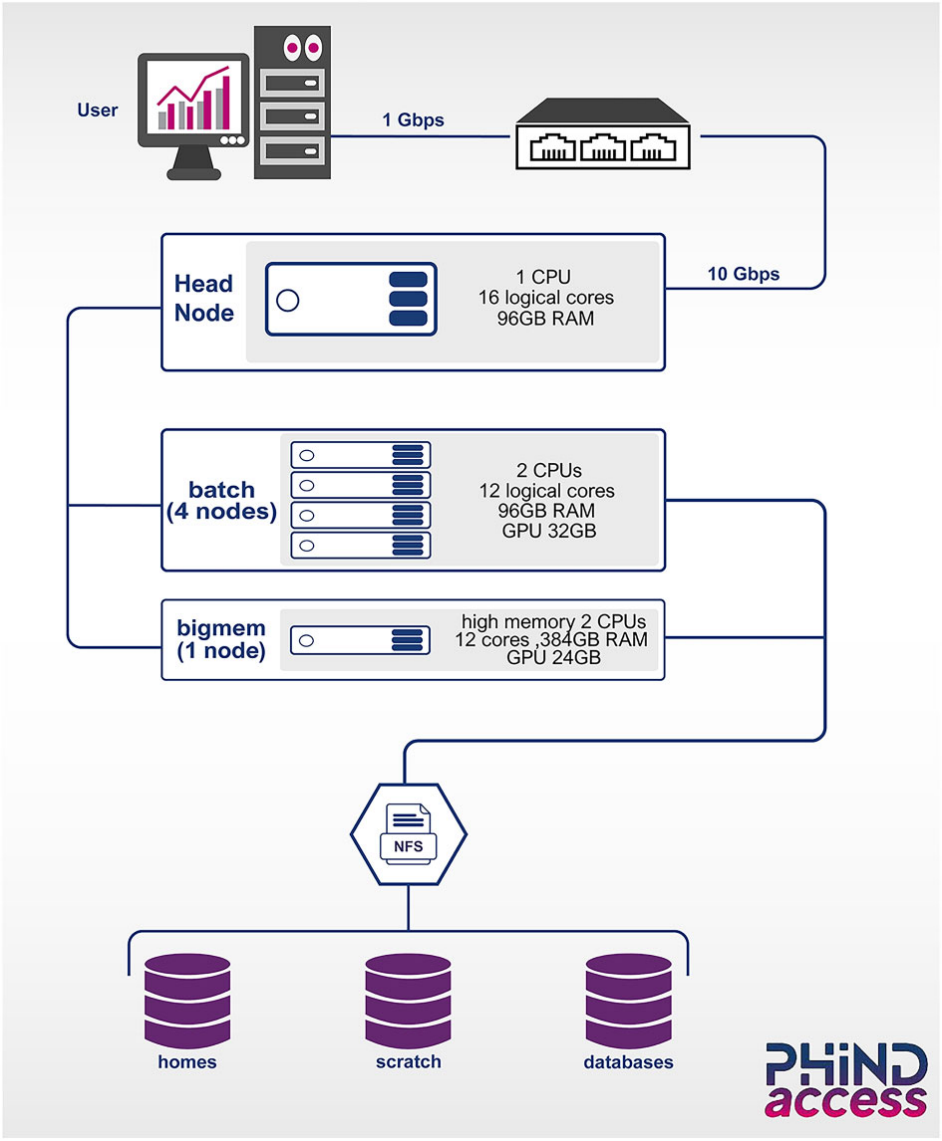
Diagram of the OMIX architecture. “*Omics*” cluster includes 1 head/login node and 5 compute nodes, including a bigmem (high memory) node. All compute nodes are equipped with GPUs. “*Omics*” consists of 272 CPU cores, 128 GPU cores, 760 GB memory, and 95 TB storage. Software packages and bioinformatics tools can be installed via Conda or through modules that are either preinstalled or can be installed upon request from the system administrator. Configuration includes a single partition for job submission, and the interconnection between login and compute nodes takes place via a 10-Gbps switch data center, featuring high throughput and low latency. “*Omics*” achieves a peak performance of 9.0 Tflops according to (LinPack benchmarks) [10].

**Figure 3:**
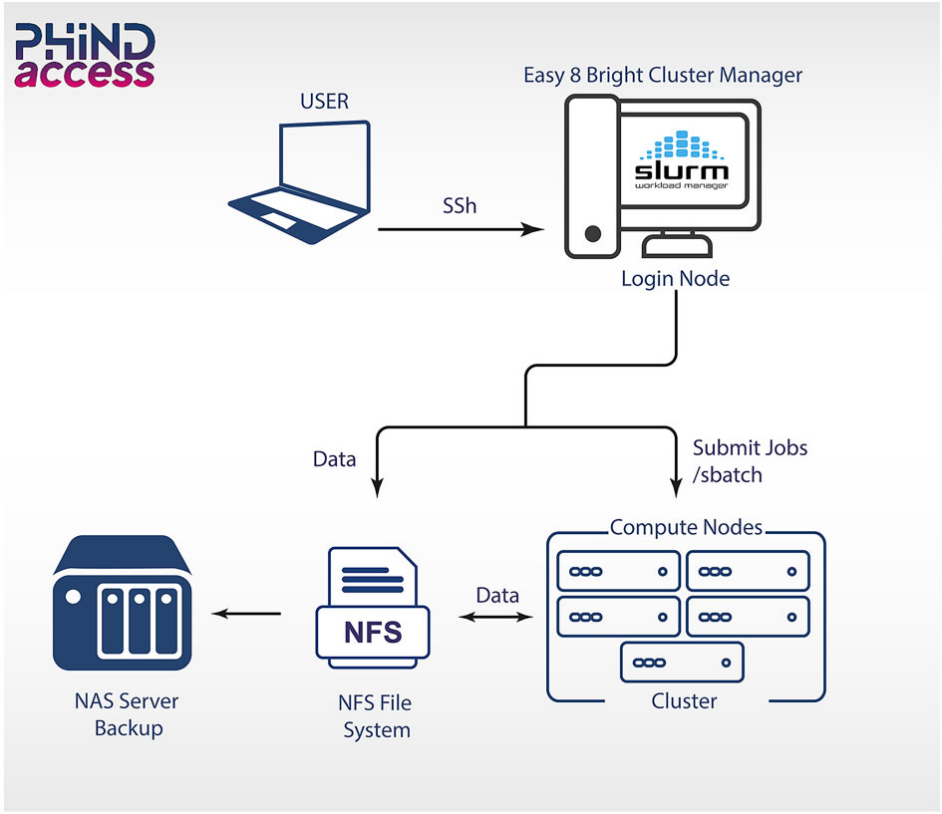
HPC cluster components and architecture. Users have to connect to the “*omics*” cluster head/login node via the SSH Linux command. The SLURM (https://slurm.schedmd.com/documentation.html) open-source cluster management system orchestrates the job scheduling and allocates resources. Jobs can be submitted and deployed on compute nodes through running bash scripting using the “sbatch” command or using the SLURM interactive mode via the “srun” command.

To identify the optimal cluster configuration, we conducted a benchmarking study, summarized in Table 1. Simplifying complexity, we implemented the NVIDIA Bright Cluster Manager v9.2 to automate setup and management of our Linux Ubuntu 20.04.6 LTS HPC cluster. Leveraging the Easy8 Bright Cluster free version, the system allows swift package installation, updates, and dependency management for various analysis types (Fig. 2). Over 60 users access the IPT’s HPC infrastructure, supported by a local Glpi Help Desk. Simple Linux Utility for Resource Management (SLURM) manages job scheduling and resource allocation (Fig. 3), while data integrity is maintained through nightly automated backups and weekly snapshots, ensuring reliable system recovery. Besides the increasing number of users, we have successfully connected our HPC cluster to multiple sequencing platforms using the server message block (SMB) protocol, enabling efficient data storage and transfer. This setup supports the management of approximately 5 TB of data generated weekly. This integration ensures a streamlined workflow and enhances our data-processing capabilities.

**Table 1:** Benchmarking of some cluster management tools based on some selected features

Features	Bright Cluster Manager	OpenHPC	Aspen Systems Cluster Management	ClusterVisor
Installation	Offers a simplified installation	Requires manual installation	Aspen provides command-line tools on the clusters for imaging, remote power, and sensor programs.	Offers a simple installation and easy accession
Configuration	Configuration process may include automated provisioning and updates.	Configuration of individual HPC software components may require a high degree of expertise.	Possibility of streamlining and configuring diverse management utilities while also handling the transfer of users’ previous utilities licenses	Use of a dashboard for an easy configuration process via the command lines and a graphical interface
Support	Typically comes with commercial support options	Community-driven with community support	Compatible with most Linux distributions and is supported for the life of the cluster	The existence and the availability of a support team
Documentation	Extensive documentation	Documentation may vary in completeness		Availability of a detailed manual
Cost	Usually involves licensing fees and support costs	OpenHPC is open source and free to use, but costs may be incurred for hardware, support, and additional software components.	Some resource manager and scheduler combinations are open source with no charges, while some are commercial products that need to be purchased.	Involves licensing fees and possibly support costs
Cluster size	Any size	Any size	Few to large groups of nodes	Any size
Monitoring	All aspects involving the cluster’s usage and stats can be easily monitored via the integrated dashboard.	All cluster stats can be monitored via ganglia, a scalable monitoring system for HPC.	All aspects of the cluster can be monitored, including performance/utilization, network saturation, power consumption, and temperature monitoring.	All aspects involving the cluster’s usage and stats can be easily monitored via the integrated dashboard.

### Training delivery

**Figure 4:**
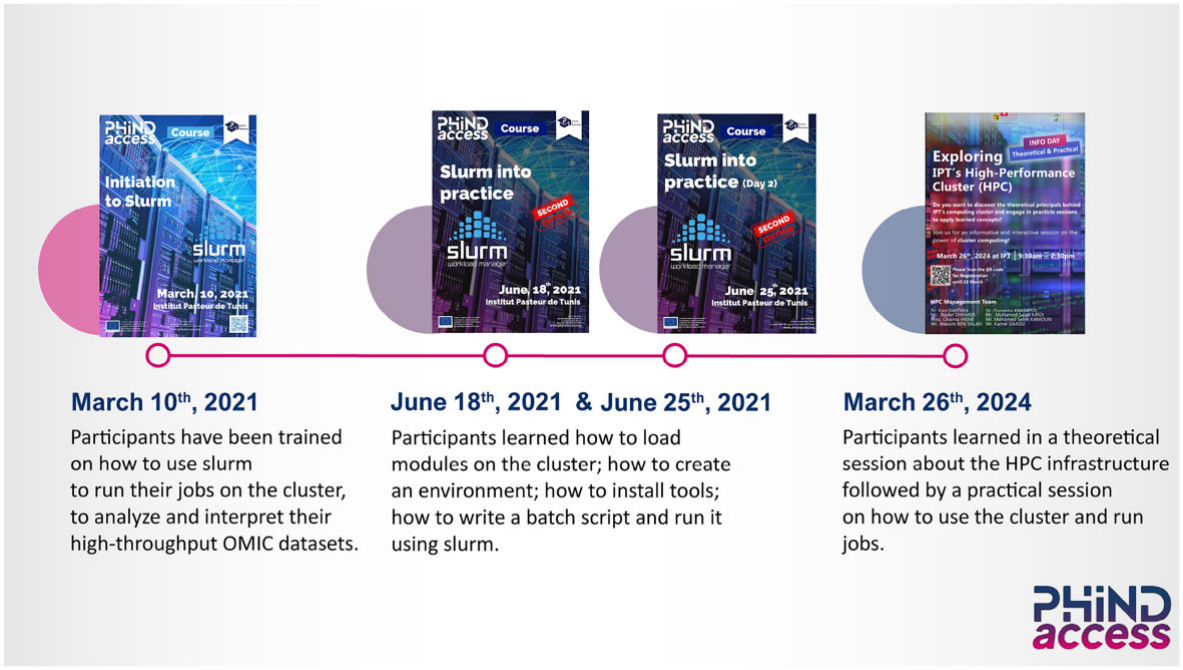
Training activities. An introductory course named “Initiation to SLURM,” followed by another course, “SLURM into Practice,” have been organized, aimed at familiarizing IPT users with the infrastructure, with bash scripting and job submission in SLURM using the “sbatch” and “srun”commands.

Training has been an integral component of the HPC facility mind-set since its launch and enables knowledge sharing across MSc and PhD students and researchers within IPT. The objective is to improve and diversify the educational component of the HPC system by providing courses on a more regular basis and targeting a wider audience (Fig. 4).

## Challenges

### Limited skilled workforce

The administration of Linux HPC systems demands highly skilled system administrators (SysAdmins) to manage the infrastructure efficiently. However, structured training programs for HPC SysAdmins are scarce and typically limited to university-level courses tailored for postgraduate computer scientists. While public resources exist for HPC user training, SysAdmins often learn through hands-on experience, which can burden HPC resources and result in inefficient management. Given the diverse institutional needs and infrastructures, a standardized training for SysAdmins is unlikely. Additionally, the lack of IT professionals skilled in Linux operating system (OS) and omics data management further complicates the issue, disrupting workflows that require expertise in Linux, command lines, and Shell/Bash scripting. Windows administrators may need to acquire Linux tools, and SysAdmins should focus on specialized tasks such as OS maintenance, user management, and data security, rather than handling all technical assistance aspects.

### Lack of onsite HPC network experts

The local IT companies lacked experience in managing this kind of infrastructure in the context of omics data analysis, thus forcing the SysAdmin to initiate the installation and configuration from scratch, which resulted in major issues. Therefore, prior familiarity with HPC architecture is crucial for SysAdmins.

## Recommendations for HPC Implementation

The challenges faced led to the development of a priority recommendations list for teams wishing to implement similar infrastructures.

### Technical recommendations

Configuring the HPC cluster is crucial to ensure its efficiency, stability, and security. Numerous software options exist; however, the best solution that we suggest, especially for smaller clusters (fewer than 8 nodes), is the Easy8 Bright Cluster Manager Solution due to its comprehensive management capabilities and free certification. Installing bioinformatics tools on an HPC cluster also poses challenges for both users and SysAdmins, particularly when different software versions are required. Thus, based on our experience, the “module” system (common on supercomputers) streamlines software usage, while Conda/Anaconda/Miniconda allows users to create Python environments and install the necessary tools efficiently.

### User training and support activities

Based on our experience, prioritizing user training in our HPC facility is a major milestone by offering comprehensive courses in SLURM and bash scripting, as this is essential for efficient job management and resource allocation (detailed in Fig. 4). Establishing detailed guidelines, standard operating procedures, and user policies is crucial for proper system use and maintenance, enhancing system efficiency and empowering researchers to actively contribute to its development and sustainability. Additionally, implementing a Google form for access requests ensures that users will demonstrate adequate knowledge of command line, SLURM, and bash scripting, further solidifying the commitment to a well-informed and capable user base.

### HPC sustainability

HPC sustainability involves ensuring the long-term viability of infrastructures based on various metrics and criteria. It encompasses human resources, infrastructure hardware, and software optimizations. Key factors include investing in specialized human resources, providing long-term contracts or permanent employment to maintain stability. Adequate funding is essential for the continuous expansion and enhancement of the infrastructure. Additionally, creating a cluster management committee is vital to knowledge sharing and documentation, keeping up with SysAdmin duties and fostering collaboration with other facilities managing HPCs in the LMICs, ensuring that the infrastructure remains efficient and effective.

## Conclusion

The IPT HPC infrastructure journey, from challenges to deployment, offers insights for LMICs. Key recommendations cover cluster configuration, management, sustainability, and user training, forming an efficient implementation framework. Such initiatives promote equitable access to computing resources, fostering global scientific collaboration and advancing scientific development.

## Abbreviations

HPC: high-performance computing; IPT: Institut Pasteur de Tunis; LMICs: low- and middle-income countries; MESRS: Tunisian Ministry of Scientific Research and Higher Education; OS: operating system; SLURM: Simple Linux Utility for Resource Management; SMB: server message block; SysAdmin: system administrator.

## Supplementary Material

giae060_GIGA-D-24-00092

giae060_GIGA-D-24-00092_R1

giae060_Response_to_Reviewer_Comments_Original_Submission

giae060_Reviewer_1_Report_Original_Submission Kary Ocana -- 4/29/2024 Reviewed

## Data Availability

Not applicable

## References

[1] Zhang Y , Cheng Y, Jia K, et al. Opportunities for computational techniques for multi-omics integrated personalized medicine. Tsinghua Sci Technol. 2014;19(6):545–58. 10.1109/TST.2014.6961025.

[2] Yu XT , Zeng T. Integrative analysis of omics big data. Methods Mol Biol. 2018;1754:109–35. 10.1007/978-1-4939-7717-8_7. 29536440

[3] Tulasi BBS , Rupali SW. High performance computing and big data analytics—paradigms and challenges. Int J Comput Appl. 2015;116(2):28–33. 10.5120/20311-2356.

[4] Leff D , Yang GZ. Big data for precision medicine. Engineering. 2015;1:277. 10.15302/J-ENG-2015075.

[5] UNESCO . UNESCO recommendation on open science. SC-PCB-SPP/2021/OS/UROS, UNESCO, 2021. 10.54677/MNMH8546.

[6] Courneya JP , Mayo A. High-performance computing service for bioinformatics and data science. J Med Library Assoc. 2018;106(4):494. 10.5195/jmla.2018.512. PMC6148605 30271293

[7] Kurc T , Hastings S, Kumar V, et al. HPC and grid computing for integrative biomedical research. Int J High Perform Comput Appl. 2009;23:252. 10.1177/1094342009106192. 20107625 PMC2811341

[8] Hkimi C , Kamoun S, Khamessi O, et al. Mycobacterium tuberculosis-THP-1 like macrophages protein-protein interaction map revealed through dual RNA-seq analysis and a computational approach. J Med Microbiol. 2024;73(2):001803. 10.1099/jmm.0.001803. 38314675

[9] Ghedira K , Dallali H, Ardhaoui M, et al. PHINDaccess hackathons for COVID-19 and host-pathogen interaction: lessons learned and recommendations for low-and middle-income countries. BioMed Res Int. 2023;2023:6638714. 10.1155/2023/6638714. 37854792 PMC10581832

[10] Dongarra J , Luszczek P, Petitet A. The LINPACK Benchmark: past, present and future. Concurr Comp Pract Exp. 2003;15:803–20. 10.1002/cpe.728.

