# Supplementary material for: Design and implementation of a scalable high-performance computing (HPC) cluster for omics data analysis: achievements, challenges and recommendations in LMICs
Source: Gigascience. 2024 Aug 22;13:giae060. doi: 10.1093/gigascience/giae060 (PMC11340639; doi:10.1093/gigascience/giae060)
Supplement: giae060_GIGA-D-24-00092 [file giae060_giga-d-24-00092.pdf]

GigaScience

Design and Implementation of a scalable High Performance Computing (HPC) Cluster for OMICS data analysis: Achievements, challenges and recommendations in LMICs

--Manuscript Draft--

Manuscript Number:	GIGA-D-24-00092
Full Title:	Design and Implementation of a scalable High Performance Computing (HPC) Cluster for OMICS data analysis: Achievements, challenges and recommendations in LMICs
Article Type:	Commentary
Funding Information:	
Abstract:	The advent of high-throughput technologies, including cutting-edge sequencing devices, has revolutionized biomedical data generation and processing. Nevertheless, big data applications require novel hardware and software for parallel computing and management to handle the ever-growing data size and analysis complexity. On-premise, High Performance Computing (HPC) is increasingly used in biomedical research for big data stewardship. In this work, we present Tunisia's first high-performance computational infrastructure for OMICS research. We highlight measurements and recommendations that may help other Low and middle income countries (LMICs) institutions eager to implement local HPC facilities for bioinformatics research and OMICS data analyses.
Corresponding Author:	Kais Ghedira<br>Institut Pasteur de Tunis<br>Tunis, Tunis TUNISIA
Corresponding Author Secondary Information:	
Corresponding Author's Institution:	Institut Pasteur de Tunis
Corresponding Author's Secondary Institution:	
First Author:	Kais Ghedira
First Author Secondary Information:	
Order of Authors:	Kais Ghedira
	Oussema Khamessi
	Chaima Hkimi
	Selim Kamoun
	Nader Dhamer
	Kamel Daassi
	Wassim Ben Salah
	Houcemeddine Othman
	Wahbi Belhadj
	Youssef Ghorbal
Order of Authors Secondary Information:	
Additional Information:	
Question	Response
Are you submitting this manuscript to a special series or article collection?	No
Experimental design and statistics	Yes

Powered by Editorial Manager® and ProduXion Manager® from Aries Systems Corporation

Full details of the experimental design and statistical methods used should be given in the Methods section, as detailed in our Minimum Standards Reporting Checklist. Information essential to interpreting the data presented should be made available in the figure legends.<br><br>Have you included all the information requested in your manuscript?	
**Resources**<br><br>A description of all resources used, including antibodies, cell lines, animals and software tools, with enough information to allow them to be uniquely identified, should be included in the Methods section. Authors are strongly encouraged to cite Research Resource Identifiers (RRIDs) for antibodies, model organisms and tools, where possible.<br><br>Have you included the information requested as detailed in our Minimum Standards Reporting Checklist?	Yes
**Availability of data and materials**<br><br>All datasets and code on which the conclusions of the paper rely must be either included in your submission or deposited in publicly available repositories (where available and ethically appropriate), referencing such data using a unique identifier in the references and in the "Availability of Data and Materials" section of your manuscript.<br><br>Have you have met the above requirement as detailed in our Minimum Standards Reporting Checklist?	Yes

Manuscript

Click here to access/download;Manuscript;Commentary_GigaScience_Jour

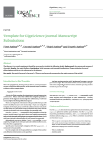

GigaScience, 2023, 1–5

doi: xx.xxxx/xxxx
Manuscript in Preparation
Paper

PAPER

Design and Implementation of a scalable High Performance Computing (HPC) Cluster for OMICS data analysis: Achievements, challenges and recommendations in LMICs

Kais Ghedira[1,*], Oussema Khamessi[2], Chaima Hkimi[1,2], Selim Kamoun[1,2], Nader Dhamer[3], Kamel Daassi[3], Wassim Ben Salah[3], Houcemeddine Othman[4], Wahbi Belhadj[5] and Youssef Ghorbal[6]

[1]Laboratory of Bioinformatics, Biomathematics and Biostatistics, LR16IPT09, Institut Pasteur de Tunis, University of Tunis El Manar, Tunis, Tunisia and [2]High Institute of Biotechnology of Sidi Thabet, University of Manouba, Ariana BP-66, Manouba 2010, Tunisia. and [3]Direction Informatique de l'Institut Pasteur de Tunis and [4]Sydney Brenner Institute for Molecular Bioscience, Faculty of Health Sciences, University of the Witwatersrand, Johannesburg, South Africa and [5]The european bioinformatics institute-European Bioinformatics Institute (EMBL-EBI) and [6]HPC Core Facility of the Institut Pasteur Paris, France

[*]Correspondance should be addressed to kais.ghedira@pasteur.tn

Abstract

Background, The advent of high-throughput technologies, including cutting-edge sequencing devices, has revolutionized biomedical data generation and processing. Nevertheless, big data applications require novel hardware and software for parallel computing and management to handle the ever-growing data size and analysis complexity. On-premise, High Performance Computing (HPC) is increasingly used in biomedical research for big data stewardship. **Findings**, In this work, we present Tunisia's first high-performance computational infrastructure for OMICS research. **Method**, We highlight measurements and recommendations that may help other Low and middle income countries (LMICs) institutions eager to implement local HPC facilities for bioinformatics research and OMICS data analyses.

Key words: Bioinformatics; HPC; Tunisia; Infrastructure; computational power; computing cluster

Background

Over the past two decades, advancements in high throughput technologies have resulted in a massive accumulation of OMICS data, driving biomedical sciences into a big data era [1, 2]. Nowadays, a number arising of sequence-based approaches are becoming common biomedical tools, leading to traditional computing approaches becoming insufficient to handle the vast data volumes. Robust high-performance computing infrastructure has now become mandatory to efficiently manage and analyze this huge amount of data as well as the integration of cutting-edge omics and artificial intelligence techniques [3, 4]. In this context, it's vital to acknowledge the disparities faced by researchers in Low and Middle-Income Countries (LMICs) regarding the access and use of such sophisticated computational resources. Indeed, open science infrastructures play a role in promoting parity among researchers from poor and developed nations by facilitating the fair and reciprocal exchange of scientific inputs and outputs. This also emphasizes the need for infrastruc-

Compiled on: March 19, 2024.
Draft manuscript prepared by the author.

Key Points

- HPC successful implementation process within LMIC, showcasing its vital role in OMICS research.
- Addressing challenges and barriers faced during the HPC implementation process in the context of LMICs.
- Effective recommendations for HPC infrastructures implementation for OMICS sciences within LMICs.

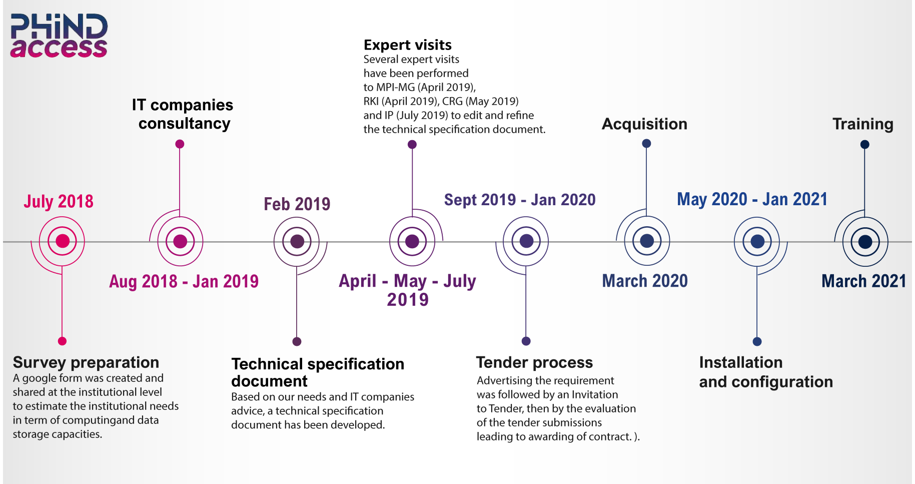

Figure 1. Steps and timeline followed for the acquisition of the infrastructure

tures offering computational and data manipulation services [5]. Previous studies have utilized Grid and HPC infrastructures for integrative biomedical research [6, 7]. Indeed, various tools have been developed for exome analysis, RNA clustering, and RNA-seq data analysis on HPC clusters [8]. Here, we discuss the implementation of an HPC infrastructure at the Institut Pasteur de Tunis (IPT) through the PHINDaccess twinning project, aiming to enhance Omics data management and analysis in the context of pathogen-host interaction studies [9].

Design of the infrastructure and Timeline

Hardware acquisition occurred in March 2020 in the frame of the twinning H2020 PHINDaccess project with funding secured through the help of the Tunisian Ministry of Scientific Research and Higher Education (MESRS). System administrators and project members collaborated to make the cluster functional. In January 2021, the HPC facility named OMICS was introduced as an institutional bioinformatics platform aiming to enhance users' capacities in OMICS data analysis and management (Figure1). A benchmarking study comparing various cluster management tools was conducted to determine the optimal configuration as summarized in Table1.

HPC configuration

To minimize complexity, the NVIDIA Bright Cluster Manager software v9.2 has been implemented to automate the setup and management of our Linux Ubuntu 20.04.6 LTS HPC cluster. Leveraging the Easy8 Bright Cluster free version, which is tailored for clusters of up to 8 non-accelerated nodes, OMICS now consists of 272CPU cores, 128GPU cores, 750GB memory, and 95TB storage. Configuration includes a single partition for job submission, (Figure2) and the interconnection between compute and login nodes takes place via a 10Gbps switch data center, featuring high throughput and low latency. OMICS achieves a peak performance of 9.0 Tflops according to LinPack benchmarks [10].

The OMICS IPT HPC Cluster Design and Implementation

The OMICS HPC facility was introduced as an institutional bioinformatics platform in 2021 (January) in order to help IPT users to improve their OMICS data analysis and data management capabilities. A benchmarking of the different existing solutions to configure and manage the HPC was performed. Table 1 summarizes the results of the benchmarking of some cluster management tools based on some important features and criterias.

Software packages and bioinformatics tools can be installed using Conda. The system allows swift package installation, updates, and dependency management for various analysis types. More than 40 users have gained access to the IPT HPC infrastructure. Assistance is provided through a local instance of the Glpi Help Desk ticketing system. SLURM, an open-source cluster management system, orchestrates job scheduling and resources allocation (Figure3). Regarding data safety and integrity, backups for omics data is ensured through a nightly automated process taping the data on an independent network-attached storage as well as a weekly snapshots system holding a copy of the entire storage at the point they were taken, thus allowing for system recovery.

Training activities

HPC facility prioritizes training, offering SLURM and bash scripting courses to users (Figure4). Guidelines, SOPs, and user policies have been set to ensure a proper system use and maintenance, thus allowing researchers to contribute to the system development and sustainability. A Google form has also been set for users in order to request access to the cluster, providing a justification and demonstrating knowledge of command line, SLURM as well as bash scripting.

Challenges

Lack of HPC SysAdmin Training

Linux HPC administration requires highly skilled SysAdmins for efficient infrastructure management. However, structured training

Table 1. Benchmarking of some cluster management tools based on some selected features

Features	Bright Cluster Manager	OpenHPC	Aspen Systems Cluster Management	ClusterVisor
Installation	Offers a simplified installation.	Requires manual installation	Aspen provides command line tools on the clusters for imaging, remote power and sensor programs	Offers a simple installation and easy accession
Configuration	Configuration process may include automated provisioning and updates.	Configuration of individual HPC software components may require a high degree of expertise.	Possibility of streamlining and configuring diverse management utilities while also handling the transfer of users previous utilities licenses.	Use of a dashboard for an easy configuration process via the command lines and a graphical interface.
Support	Typically comes with commercial support options.	Community-driven with community support	Compatible with most Linux distributions and is supported for the life of the cluster	Disponibility of a support team
Documentation	Extensive documentation	Documentation may vary in completeness		Availability of a detailed manual
Cost	Usually involves licensing fees and support costs	OpenHpc is open-source and free to use, but costs may be incurred for hardware, support and additional software components	Some resource manager and scheduler combinations are open source with no charges, while some are commercial products that need to be purchased.	Involves licensing fees and possibly support costs
Cluster size	Any size	Any size	Few to large groups of nodes.	Any size
Monitoring	All aspects involving the cluster's usage and stats can be easily monitored via the integrated dashboard	All cluster stats can be monitored via ganglia, a scalable monitoring system for HPC.	All aspects of the cluster can be monitored, including performance/utilization, network saturation, power consumption, temperature monitoring.	All aspects involving the cluster's usage and stats can be easily monitored via the integrated dashboard

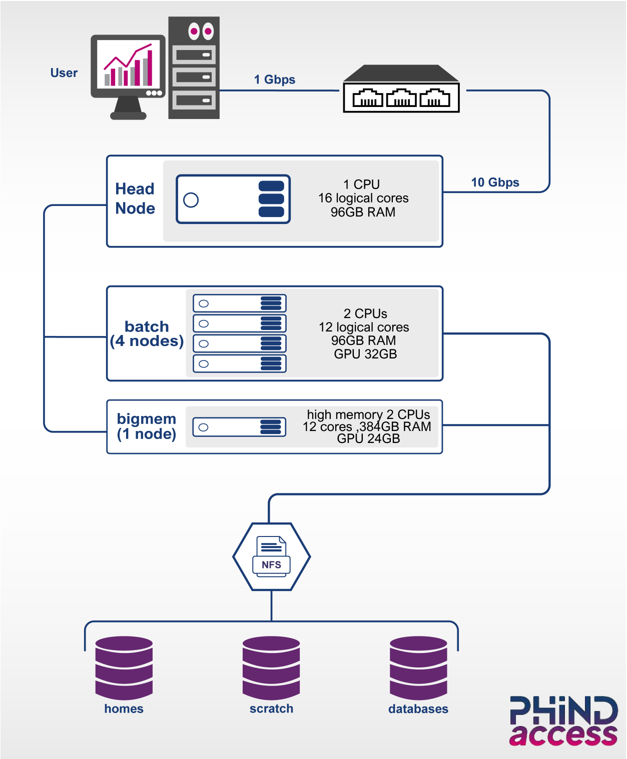

Figure 2. Diagram of the OMIX architecture

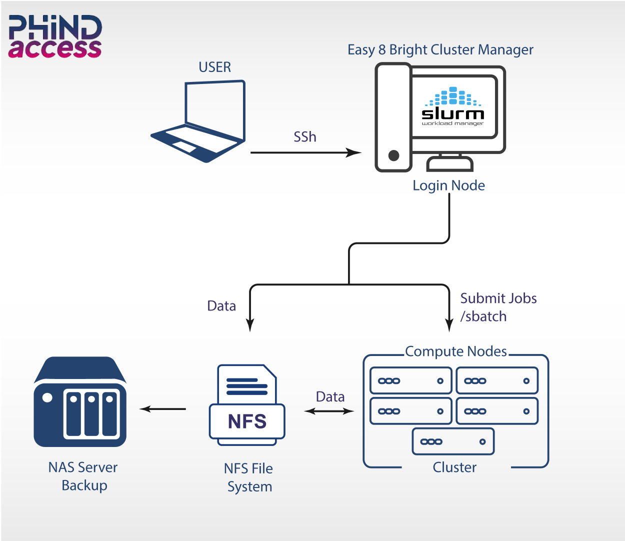

Figure 3. HPC cluster components and architecture

programs for HPC SysAdmins are scarce, often limited to university-level courses tailored for postgraduate computer scientists. While public resources exist for HPC users training, SysAdmins usually learn by doing. This approach can burden HPC resources and lead to inefficient management. Because institutional needs and infrastructures are diverse, a standardized training for SysAdmins is unlikely to be achievable. Thus, the development of a thorough HPC SysAdmin curriculum is crucial in order to face these challenges.

Lack of Skilled IT Human Resources in Linux

A lack of IT human resources skilled in Linux OS and omics data management can disrupt workflows. Indeed, managing multi-omics big data requires expertise in Linux, command lines, and Shell/Bash scripting. In that regard, Windows administrators may need to learn Linux tools. SysAdmins should also mainly focus on specialized tasks such as OS maintenance, user management and data security, among other things rather than handling all technical

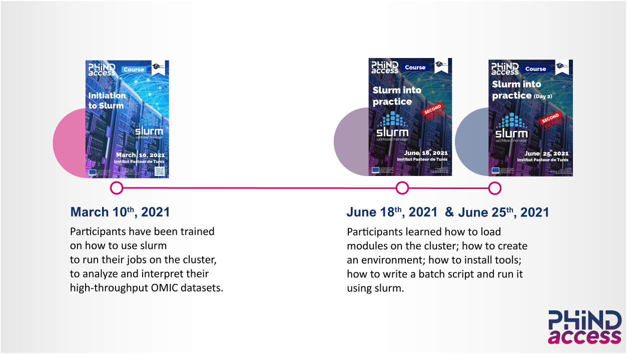

Figure 4. Training activities

assistance aspects.

Lack of onsite HPC network experts
The local company lacked experience in managing this kind of infrastructure in the context of OMICS data analysis, thus forcing the SysAdmin to start the installation and configuration from scratch, which resulted in major issues. Therefore, prior familiarity with HPC architecture is crucial for SysAdmins.

Recommendations for HPC Implementation

The challenges faced led to the development of a priority recommendations list for teams wishing to implement similar infrastructures.

Cluster Configuration
Configuring the HPC cluster is crucial to ensure its efficiency, stability, and security. Numerous software options exist. However the best solution, especially for smaller clusters (less than 8 nodes), is Easy8 Bright Cluster Manager Solution for its comprehensive management and free certification.

User Training
Training users on HPC schedulers is essential for efficient job management. Schedulers control job queues, prioritize and distribute jobs, and allocate resources. Thus, users need prior familiarization and training on scheduler usage.

Bioinformatics Tools Installation
Installing bioinformatics tools on an HPC cluster poses challenges for both users and SysAdmins. Conflicts arise, particularly when users require different software versions. The "module" system, common on supercomputers, streamlines software usage. Conda/Anaconda/Miniconda allows users to create Python environments and install required tools efficiently.

HPC Sustainability
HPC sustainability involves ensuring the long-term viability of infrastructures based on various metrics and criteria. It encompasses human resources, infrastructure hardware and machines. Key factors for infrastructure sustainability include investing in specialized human resources, providing long-term contracts or permanent employment as well as allocating sufficient funding for the infrastructure's expansion. Another vital point would be scheduling regular meetings between IT centers to keep up with SysAdmin duties and strengthen collaborations.

Conclusion

The IPT HPC infrastructure journey, from challenges to deployment, offers insights for LMICs. Key recommendations cover cluster configuration, management, sustainability, and user training, forming an efficient implementation framework. Such initiatives promote equitable access to computing resources, fostering global scientific collaboration and advancing scientific development.

Data Availability

Not applicable

Conflicts of Interest

The authors declare that there is no conflict of interest regarding the publication of this paper.

Supplementary Materials
Additional file 1: PhindAccess_survey.pdf

List of abbreviations

HPC: High Performance Computing; IPT: Institut Pasteur de Tunis; LMICs: Low and middle income countries; MESRS: Tunisian Ministry of Scientific Research and Higher Education; SLURM: Simple Linux Utility for Resource Management

Ethical Approval (optional)

'Not applicable'.

Consent for publication

'Not applicable'

Competing Interests

'The author(s) declare that they have no competing interests'.

Funding

This work was supported by the Tunisian Ministry of Higher Education and Scientific Research and the Institut Pasteur de Tunis. The PHINDaccess bioinformatics infrastructure has been funded by the Tunisian Ministry of Higher Education and Scientific Research. The study was supported by the European project PHINDaccess: Strengthening Omics data analysis capacities in pathogen-host interaction (Grant agreement ID: 811034).

Author's Contributions

Kais Ghedira: Designed and supervised the study, wrote the manuscript, reviewed the manuscript
Oussema Khamessi, Chaima Hkimi, Selim Kamoun: reviewed the manuscript, worked on the figures generation
Nader Dhamer, Wahbi Belhadj, Youssef Ghorbal: Implemented and configured the HPC cluster, wrote the manuscript
Youssef Ghorbal: Supervised the study, Implemented and configured the HPC cluster, wrote the manuscript
Kamel Daassi, Wassim Ben Salah, Houcemeddine Othman: contributed in the writing of the manuscript

Acknowledgements

We would like to acknowledge Pr Samia Menif, General Director of the Institut Pasteur de Tunis as well as Pr Helmi Mardassi, the

Principal Investigator of the PHINDaccess project and the Tunisian Ministry of higher education and scientific research (MESRS) for funding the acquisition of the bioinformatics infrastructure. We also acknowledge the help of the HPC Core Facility of the Institut Pasteur for this work.

Authors' information (optional)

You may choose to use this section to include any relevant information about the author(s) that may aid the reader's interpretation of the article, and understand the standpoint of the author(s). This may include details about the authors' qualifications, current positions they hold at institutions or societies, or any other relevant background information. Please refer to authors using their initials. Note this section should not be used to describe any competing interests.

References

1. Zhang Y, Cheng Y, Jia K, Zhang A. Opportunities for computational techniques for multi-omics integrated personalized medicine. Tsinghua Science and Technology 2014;19(6):545–558.
2. Yu XT, Zeng T. Integrative analysis of omics big data. Computational Systems Biology: Methods and Protocols 2018;p. 109–135.
3. Tulasi B BS Rupali Sunil Wagh. High Performance Computing and Big Data Analytics - Paradigms and Challenges. International Journal of Computer Applications 2015 April;116(2):28–33. https://ijcaonline.org/archives/volume116/number2/20311-2356/.
4. Leff D, Yang GZ. Big Data for Precision Medicine. Engineering 2015 09;1:277.
5. UNESCO. UNESCO Recommendation on Open Science 2021;https://unesdoc.unesco.org/ark:/48223/pf0000379949.
6. Courneya JP, Mayo A. High-performance computing service for bioinformatics and data science. Journal of the Medical Library Association: JMLA 2018;106(4):494.
7. Kurc T, Hastings S, Kumar V, Langella S, Sharma A, Pan T, et al. HPC and Grid Computing for Integrative Biomedical Research. The international journal of high performance computing applications 2009 08;23:252.
8. Kawalia A, Motameny S, Wonczak S, Thiele H, Nieroda L, Jabbari K, et al. Leveraging the power of high performance computing for next generation sequencing data analysis: tricks and twists from a high throughput exome workflow. PloS one 2015;10(5):e0126321.
9. Ghedira K, Dallali H, Ardhaoui M, Bouslema Z, Hamdi Y, Feki Ben-Salah S, et al. PHINDaccess Hackathons for COVID-19 and Host-Pathogen Interaction: Lessons Learned and Recommendations for Low-and Middle-Income Countries. BioMed Research International 2023;2023.
10. Dongarra J, Luszczek P, Petitet A. The LINPACK Benchmark: past, present and future. Concurrency and Computation: Practice and Experience 2003 08;15:803–820.

Figure

Click here to access/download;Figure;Figure 1.tif

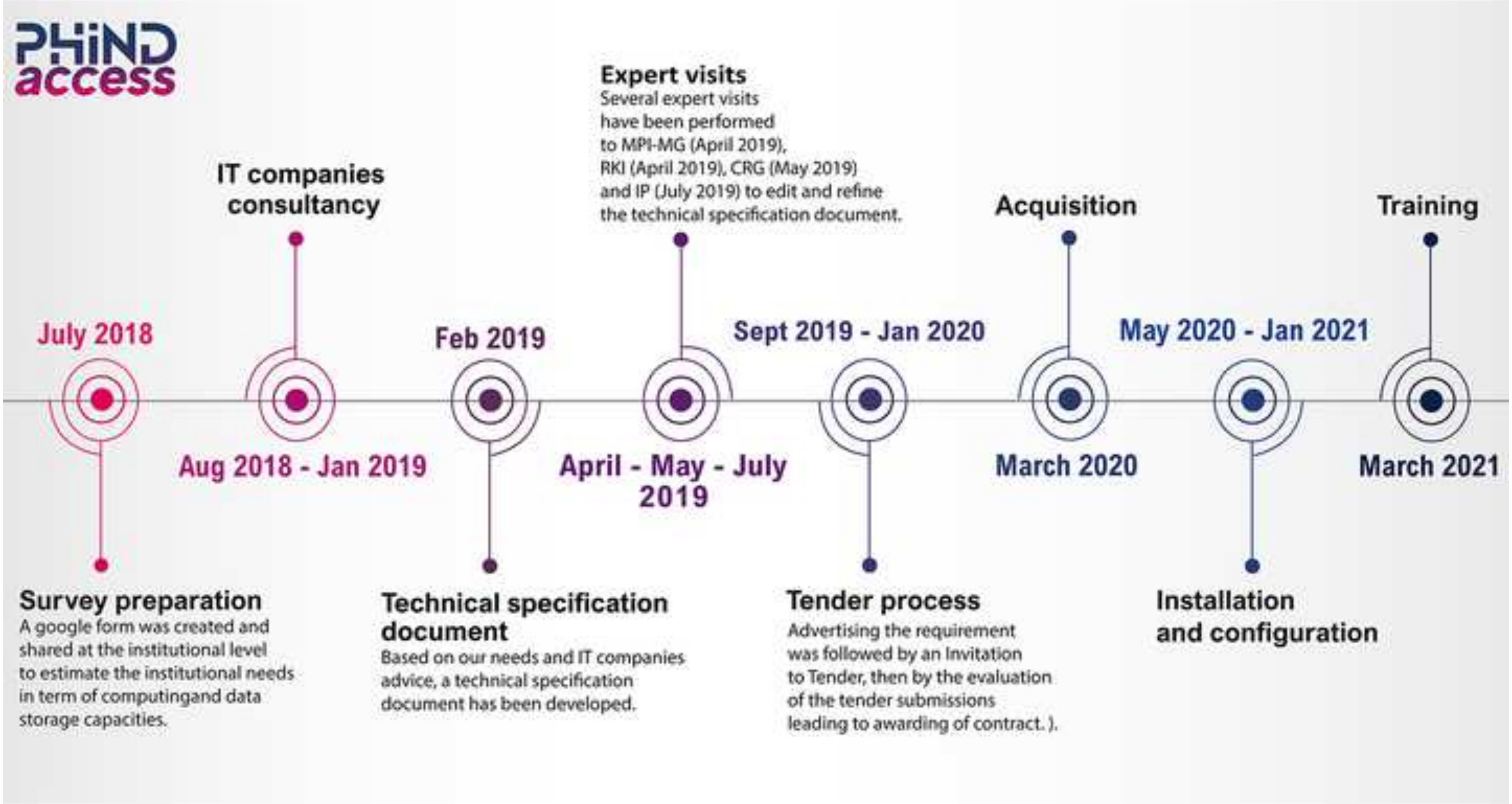

 Click here to access/download;Figure;Figure 2.tif

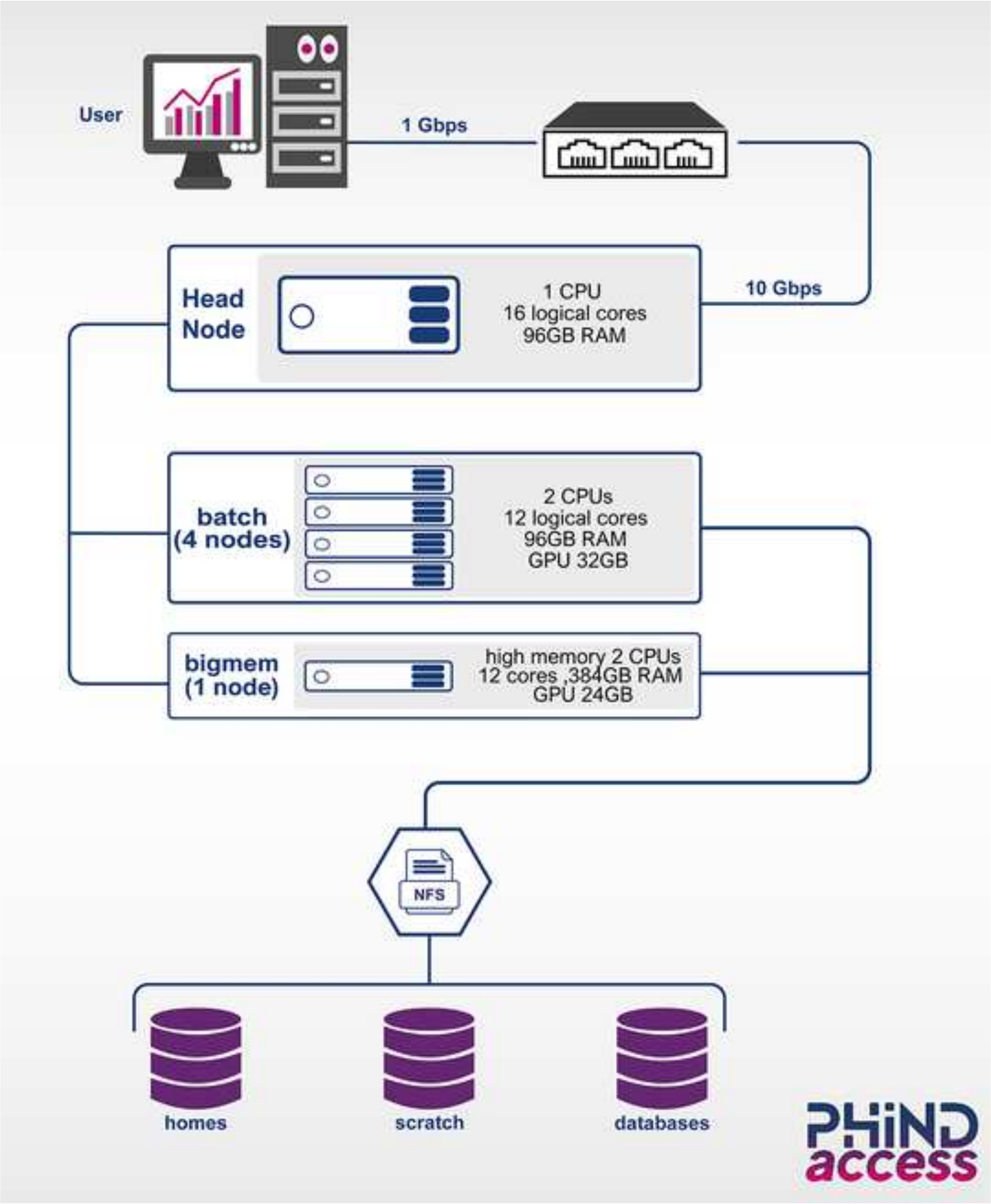

Figure

Click here to access/download;Figure;Figure 3.tif

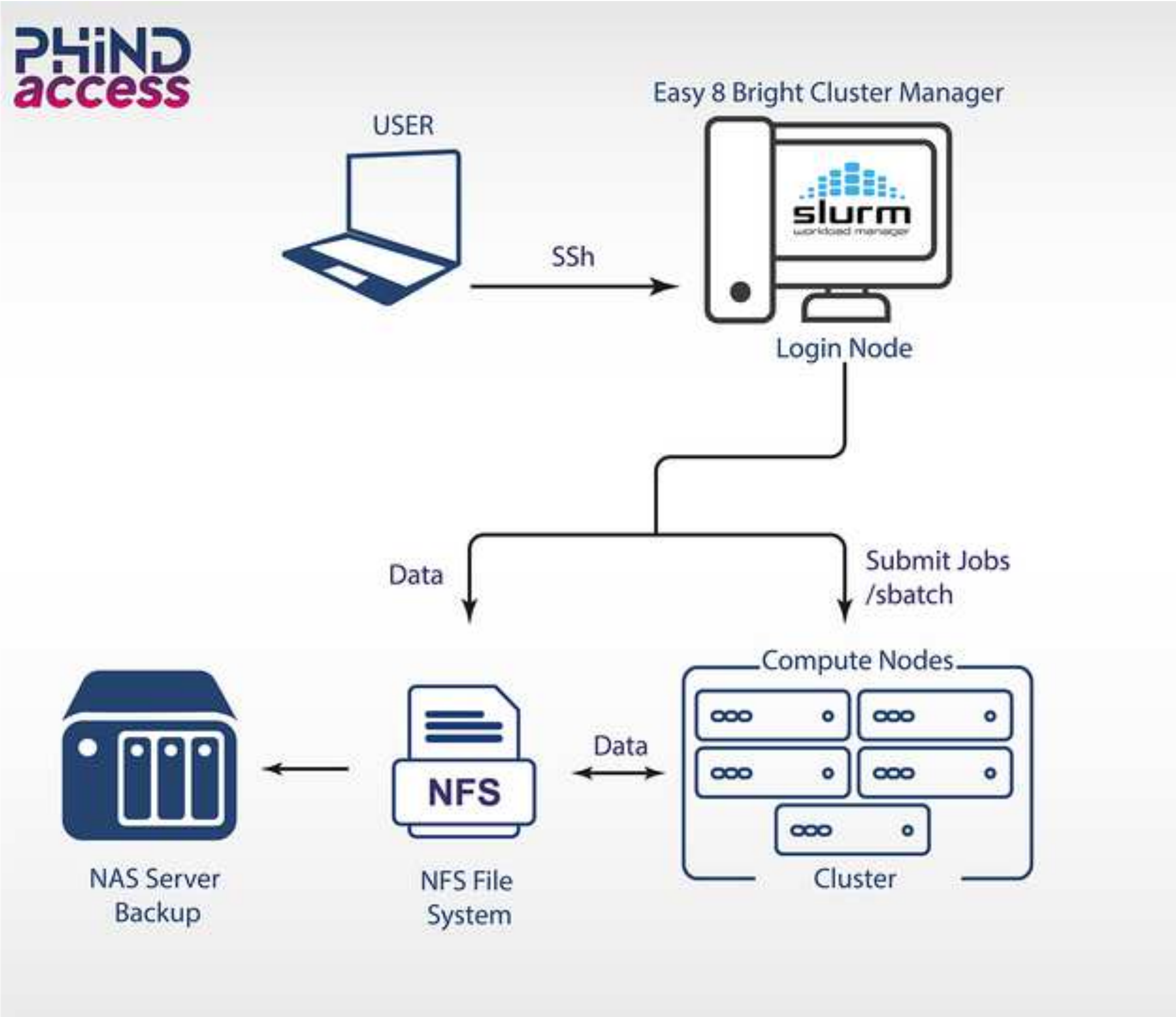

Click here to access/download;Figure;Figure 4.tif

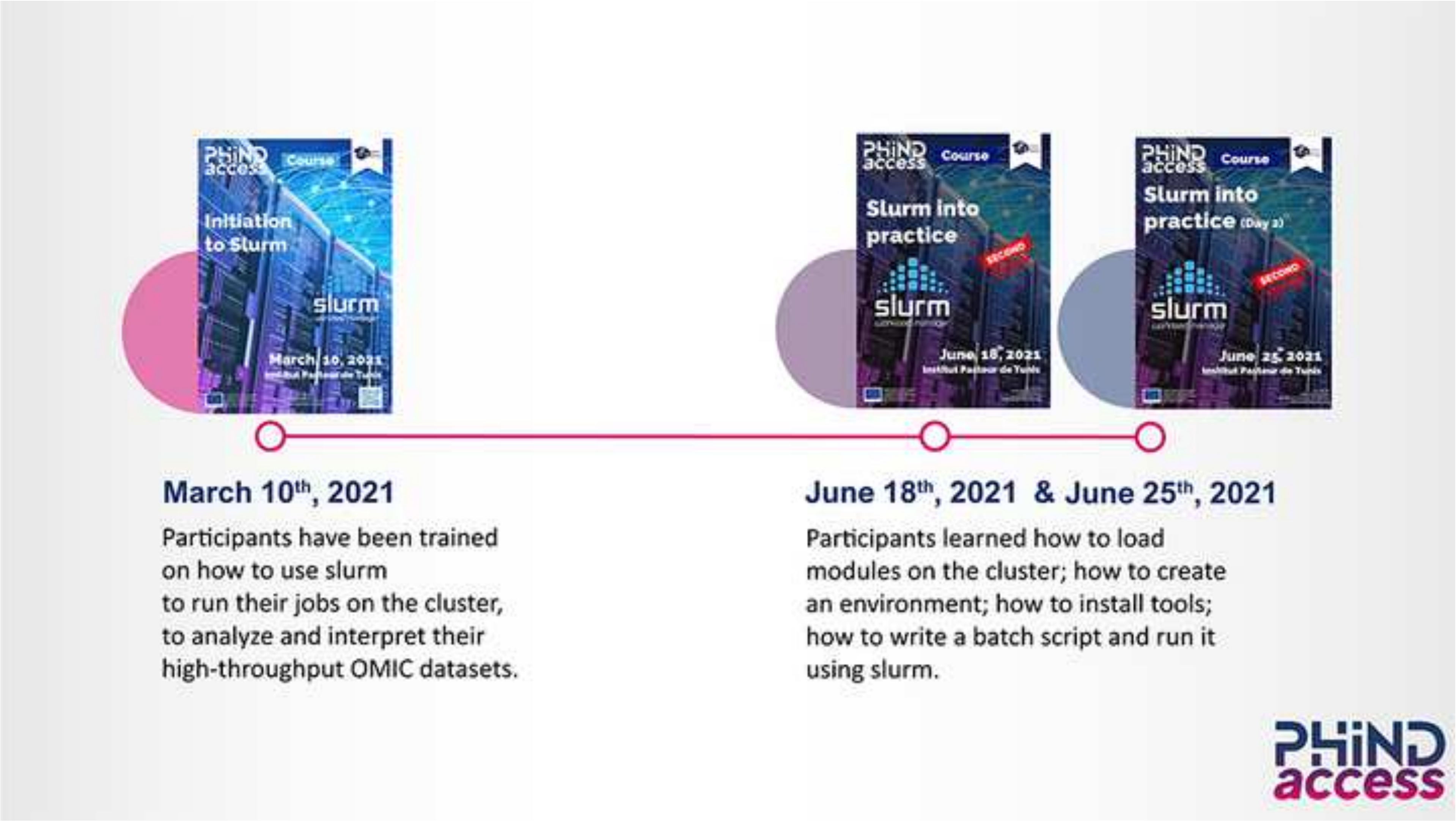

Personal Cover Click here to access/download;Personal Cover;cover letter gigascience.pdf

Kais Ghedira
13, Place Pasteur B.P.74
Tunis, 1002, Tunisia.
+216 53 370 404
kais.ghedira@pasteur.tn

Dear Editors,

We are pleased to address this letter to you to inquire about the possibility of publishing our article entitled: "Design and Implementation of a scalable High Performance Computing (HPC) Cluster for OMICS data analysis: Achievements, challenges and recommendations in LMICs" by Kais Ghedira et al in your highly esteemed *GigaScience* journal as a commentary article.

This paper outlines the steps undertaken in order to implement an operational HPC cluster at the Institut Pasteur de Tunis, Tunisia. We also highlight the difficulties and challenges faced during the whole process as well as recommendations that may prove to be useful to other organizations and institutions in LMICs that are keen to set up local HPC facilities to help enhance bioinformatics analysis.

We chose to submit this article as a commentary based on the recommendations in response to our Presubmission enquiry to GigaScience journal. Indeed, after submission and after reading the manuscript draft, the editor recommended that we could consider submitting it as a commentary, as it would fit more in that category, provided that we make it shorter. We proceeded with the necessary modifications based on the editor's recommendations and remarks. The details of the discussion follows:

“Dear Prof Kais,

Many thanks for your enquiry to GigaScience. Reading your draft it sounds potentially interesting, although the article type for such a piece is a bit tricky to pin down as its more a narrative Review or Commentary rather than Research or Software article. We do take some of these, but in its current form its a little long for a Commentary, and a little short for a Review. If you were able to trim it down to ~1500 words and 10 references, focussing on the recommendations it could fit as a Commentary. And if you were able to expand upon a the background and discussion a bit to really increase the context and relevance to data intensive research in LMICs it could be considered as a Review article.

If you wanted to do that I've annotated some feedback on the PDF, and can suggest the following additions (it doesn't need to be that much longer, but this would improve the introduction and conclusions).

The title highlights the importance to LMICs, but the introduction doesn’t explain this. Therefore in the background could you maybe add a little more context on the challenges for LMIC countries in participating in data intensive research. Maybe putting it in the context and cite the UNESCO Open Science Recommendation (https://doi.org/10.54677/MNMH8546) which says that open science infrastructures are a key pillar “ensuring equity among researchers from developed and developing countries, enabling fair and reciprocal sharing of scientific inputs and outputs”. This also highlights the need for “computational and data

manipulation service infrastructures that enable collaborative and multidisciplinary data analysis and digital infrastructures"

In one of the figures you also show the IPT network links to H3ABioNet/H3Africa but you don't expand upon this anywhere in the paper, so can you add a bit more on this? What support and advantages do these regional networks provide, and obviously funding is a major limiting issue here so do they or any other global funders offer opportunities?

You've also got sections on training that don't provide a lot of examples or resources that could be expanded on. The recommendations at the end are the most useful and important part, but the paper suddenly just ends after that, so could you add a concluding paragraph reiterating the key points and putting them in context of take home lessons for other groups in LMICs wanting to set up HPC systems like yours. Also now the system is up and running, maybe it would be good to provide some brief examples of successes and outputs researchers have now created with the infrastructure.

I hope this is useful and let me know if you want to revise and submit, and I'm happy to look at another draft. If you do decide to submit, then please mention this discussion in the covering letter.

Best wishes,

Scott
"

We believe that our work here is eligible for publication in *GigaScience* Indeed, on premise HPC (high-performance computing) clusters are essential today in bioinformatics, multi OMICS data analysis, IA and the handling and processing of big data generated from Omics high-throughput sequencing technologies.

Regarding the funds, the presented implementation of the HPC bioinformatics infrastructure was supported by the Tunisian Ministry of Higher Education and Scientific Research and the Institut Pasteur de Tunis. The study was also supported by the European H2020 project entitled PHINDaccess: Strengthening Omics data analysis capacities in pathogen-host interaction (Grant agreement ID: 811034).

This manuscript has not been published and is not under consideration for publication elsewhere. We have no conflicts of interest to disclose.

Thank you for your consideration!
Sincerely,

The corresponding author; Dr. Kais Ghedira, Institut Pasteur de Tunis
Laboratory of Bioinformatics, Biomathematics and Biostatistics (LR20IPT09), Pasteur Institute of Tunis, University of Tunis El Manar, Tunis, 1002, Tunisia.
Email: Kais.ghedira@pasteur.tn
