# Supplementary material for: Design and implementation of a scalable high-performance computing (HPC) cluster for omics data analysis: achievements, challenges and recommendations in LMICs
Source: Gigascience. 2024 Aug 22;13:giae060. doi: 10.1093/gigascience/giae060 (PMC11340639; doi:10.1093/gigascience/giae060)
Supplement: giae060_GIGA-D-24-00092_R1 [file giae060_giga-d-24-00092_r1.pdf]

GigaScience

Design and Implementation of a scalable High Performance Computing (HPC) Cluster for OMICS data analysis: Achievements, challenges and recommendations in LMICs

--Manuscript Draft--

**Manuscript Number:**	GIGA-D-24-00092R1
**Full Title:**	Design and Implementation of a scalable High Performance Computing (HPC) Cluster for OMICS data analysis: Achievements, challenges and recommendations in LMICs
**Article Type:**	Commentary
**Funding Information:**	
**Abstract:**	Abstract: The advent of high-throughput technologies, including cutting-edge sequencing devices, has revolutionized biomedical data generation and processing. Nevertheless, big data applications require novel hardware and software for parallel computing and management to handle the ever-growing data size and analysis complexity. On-premise, High Performance Computing (HPC) is increasingly used in biomedical research for big data stewardship. In this work, we present Tunisia's first high-performance computational infrastructure for OMICS research. We highlight measurements and recommendations that may help other Low and middle income countries (LMICs) institutions eager to implement local HPC facilities for bioinformatics research and OMICS data analyses.
**Corresponding Author:**	Kais Ghedira<br>Institut Pasteur de Tunis<br>Tunis, Tunis TUNISIA
**Corresponding Author Secondary Information:**	
**Corresponding Author's Institution:**	Institut Pasteur de Tunis
**Corresponding Author's Secondary Institution:**	
**First Author:**	Kais Ghedira
**First Author Secondary Information:**	
**Order of Authors:**	Kais Ghedira
	Oussema Khamessi
	Chaima Hkimi
	Selim Kamoun
	Nader Dhamer
	Kamel Daassi
	Wassim Ben Salah
	Houcemeddine Othman
	Wahbi Belhadj
	Youssef Ghorbal
**Order of Authors Secondary Information:**	
**Response to Reviewers:**	Response to the editor and the reviewers<br><br>Having had a look at the reviewer's suggestions, we feel that it is too much for a Commentary - the reviewer would like more information added, but we suggest to keep it to our shorter Commentary format.<br><br>However, the following revisions must be made:

Powered by Editorial Manager® and ProduXion Manager® from Aries Systems Corporation

- Please update the writing to read less like a tutorial and to read more of the lessons learned - this is what we did, this is how we did it, and this is what we've learnt. Presenting it in this way will be more useful for the greater community.
-We thank the editor for this important comment. We took it into account and tried to update the writing style within the text in order to appear more like learned lessons and less like a tutorial as initially proposed.

- Please update the figure descriptions as per the Reviewer's comments and also Table 1.
Try and include as much information as possible without having to add more references (as we have a strict Reference maximum of 10 for Commentaries).
-We appreciate the editor’s suggestion. The figure descriptions have been updated as well as table1 in accordance with the suggestions. References have also been kept at a maximum of 10.

Reviewer reports:

Reviewer #1: Design and Implementation of a scalable High Performance Computing (HPC) Cluster for OMICS data analysis: Achievements, challenges and recommendations in LMICs

The paper introduces a comprehensive exploration of the design and implementation of an HPC cluster tailored for OMICS data analysis, particularly focusing on its application within Low- and Middle-Income Countries (LMICs). This topic holds relevance for both the HPC and bioinformatics communities. While the paper delves into specialized HPC technologies crucial for real-world bioinformatics studies, it lacks clarity in its objectives and could better address the aspects of achievements, challenges, and recommendations specific to LMICs. Additionally, the organization of paragraphs requires refinement, as the current presentation leans towards a tutorial-style description rather than a concise scientific review or commentary.
-We thank the reviewer for this valuable comment. The objectives and the organization of paragraphs has been addressed and updated according to the reviewer comments.

The authors' objective is to elucidate the insights and hurdles encountered during the deployment of a cluster dedicated to OMICS data analysis, aiming to provide valuable guidance for LMICs grappling with similar challenges. Although the paper briefly touches on related topics such as artificial intelligence, big data, data science, and open science infrastructure, a clearer integration of these elements into the HPC environment is warranted. Notably, Figure 1 outlines the timeline of infrastructure acquisition spanning from 2018 to 2021; however, considering the elapsed time since then (now April 2024), potential updates or changes to the environment should be acknowledged. Furthermore, incorporating experiences from other LMIC HPC centers into the discussion could enrich the paper. It is recommended to update references to include more recent and pertinent sources.
-We thank the reviewer for this comment. Figure 1 has been updated and details regarding the updates related to the environment have been added in HPC configuration section : “Till today, we have successfully connected our HPC cluster to multiple sequencing platforms using the SMB protocol, enabling efficient data storage and transfer. This setup supports the management of approximately 5 TB of data generated weekly. This integration ensures a streamlined workflow and enhances our data processing capabilities.”
Regarding the following point addressing the importance of incorporating other LMIC HPC centers experience into the discussion, as to our knowledge, there isn’t detailed and specific works treating the design and implementation of such infrastructures within research facilities in LMIC countries. This makes it hard to incorporate such aspects within this work.

Moreover, enhancing the clarity of experimental and comparative benchmarks mentioned in Table 1 would be beneficial. Additionally, the scarcity of human resources proficient in Linux and OMICS data analysis is highlighted, underscoring the interplay between computer science and bioinformatics in maintaining OMICS infrastructure. Figures 2, 3, and 4 require more detailed descriptions within the text, elucidating their

	significance, particularly regarding the relevance of Figure 4 to the paper's objectives.<br>-We thank the reviewer for this important suggestion. Table 1 has been reviewed in that regard. The suggested additions regarding the figures 2, 3, and 4 have been taken into account. However, in order to also take into account the editor's comment who suggested to not extend more the text so it would still fit the commentary format, the details suggested by the reviewer have been added into the figures description section rather than within the text. This enables us to make the reviewers suggested adjustments while still taking into account the editor's comments.<br><br>Recommendations for HPC Implementation:<br>To augment the paper's impact, the authors should provide a concrete and specific description of a bioinformatics case study, moving beyond the current generalized approach. The current presentation resembles a manual or tutorial, lacking the depth required for a scientific discourse. This adjustment would elevate the paper's value by offering practical insights derived from real-world applications.<br>-We appreciate the reviewer's suggestion. We added 2 examples in that regard. The first is one of our recent studies involving the prediction of protein-protein interactions through dual RNA-seq analysis in the context of Mycobacterium tuberculosis infection (reference 8 in the manuscript) that used this infrastructure. The second one is a hackathon organized in the frame of the twinning project PHINDaccess aiming to develop portable and reproducible genomics analysis pipelines for SARS-CoV-2 and that was made possible thanks to this infrastructure.
**Additional Information:**	
**Question**	**Response**
Are you submitting this manuscript to a special series or article collection?	No
**Experimental design and statistics**<br><br>Full details of the experimental design and statistical methods used should be given in the Methods section, as detailed in our Minimum Standards Reporting Checklist. Information essential to interpreting the data presented should be made available in the figure legends.<br><br>Have you included all the information requested in your manuscript?	Yes
**Resources**<br><br>A description of all resources used, including antibodies, cell lines, animals and software tools, with enough information to allow them to be uniquely identified, should be included in the Methods section. Authors are strongly encouraged to cite Research Resource Identifiers (RRIDs) for antibodies, model organisms and tools, where possible.	Yes

Powered by Editorial Manager® and ProduXion Manager® from Aries Systems Corporation

Have you included the information requested as detailed in our Minimum Standards Reporting Checklist?	
**Availability of data and materials**<br><br>All datasets and code on which the conclusions of the paper rely must be either included in your submission or deposited in publicly available repositories (where available and ethically appropriate), referencing such data using a unique identifier in the references and in the “Availability of Data and Materials” section of your manuscript.<br><br>Have you have met the above requirement as detailed in our Minimum Standards Reporting Checklist?	Yes

Powered by Editorial Manager® and ProduXion Manager® from Aries Systems Corporation

Manuscript

Click here to access/download;Manuscript;Manuscript_Submissions_July22

GigaScience, 2023, 1–5

doi: xx.xxxx/xxxx
Manuscript in Preparation
Paper

PAPER

Design and Implementation of a scalable High Performance Computing (HPC) Cluster for OMICS data analysis: Achievements, challenges and recommendations in LMICs

Kais Ghedira[1,*], Oussema Khamessi[1,2], Chaima Hkimi[1,2], Selim Kamoun[1,2], Nader Dhamer[1,3], Kamel Daassi[3], Wassim Ben Salah[3], Houcemeddine Othman[4], Wahbi Belhadj[5] and Youssef Ghorbal[6]

[1]Laboratory of Bioinformatics, Biomathematics and Biostatistics, LR16IPT09, Institut Pasteur de Tunis, University of Tunis El Manar, Tunis, Tunisia and [2]High Institute of Biotechnology of Sidi Thabet, University of Manouba, Ariana BP-66, Manouba 2010, Tunisia. and [3]Direction Informatique de l'Institut Pasteur de Tunis and [4]Sydney Brenner Institute for Molecular Bioscience, Faculty of Health Sciences, University of the Witwatersrand, Johannesburg, South Africa and [5]The european bioinformatics institute-European Bioinformatics Institute (EMBL-EBI) and [6]HPC Core Facility of the Institut Pasteur Paris, France

[*]Correspondance should be addressed to kais.ghedira@pasteur.tn

Abstract

Background, The advent of high-throughput technologies, including cutting-edge sequencing devices, has revolutionized biomedical data generation and processing. Nevertheless, big data applications require novel hardware and software for parallel computing and management to handle the ever-growing data size and analysis complexity. On-premise, High Performance Computing (HPC) is increasingly used in biomedical research for big data stewardship. **Findings**, In this work, we present Tunisia's first high-performance computational infrastructure for OMICS research. **Method**, We highlight measurements and recommendations that may help other Low and middle income countries (LMICs) institutions eager to implement local HPC facilities for bioinformatics research and OMICS data analyses.

Key words: Bioinformatics, HPC, Tunisia, Infrastructure, computational power, computing cluster

Background

Over the past two decades, advancements in high throughput technologies have resulted in a massive accumulation of omics data, driving biomedical sciences into a big data era [1, 2]. Consequently, robust high-performance computing (HPC) infrastructure has become mandatory to efficiently manage and analyze this huge amount of data and to integrate cutting-edge omics and artificial intelligence techniques [3, 4]. In this context, it is vital to acknowledge the disparities faced by researchers in Low and Middle-Income Countries (LMICs) regarding the access and use of such sophisticated computational resources. Open science infrastructures can promote parity among researchers from both poor and developed nations by facilitating the fair and reciprocal exchange of scientific inputs and outputs, emphasizing the need for infrastructures offering computational and data manipulation services [5]. Previous studies have demonstrated the utility of Grid and HPC infrastructures for integrative biomedical research [6, 7]. It is indeed the

Compiled on: July 22, 2024.
Draft manuscript prepared by the author.

Key Points

- HPC successful implementation process within LMIC, showcasing its vital role in OMICS research.
- Addressing challenges and barriers faced during the HPC implementation process in the context of LMICs.
- Effective recommendations for HPC infrastructures implementation for OMICS sciences within LMICs.

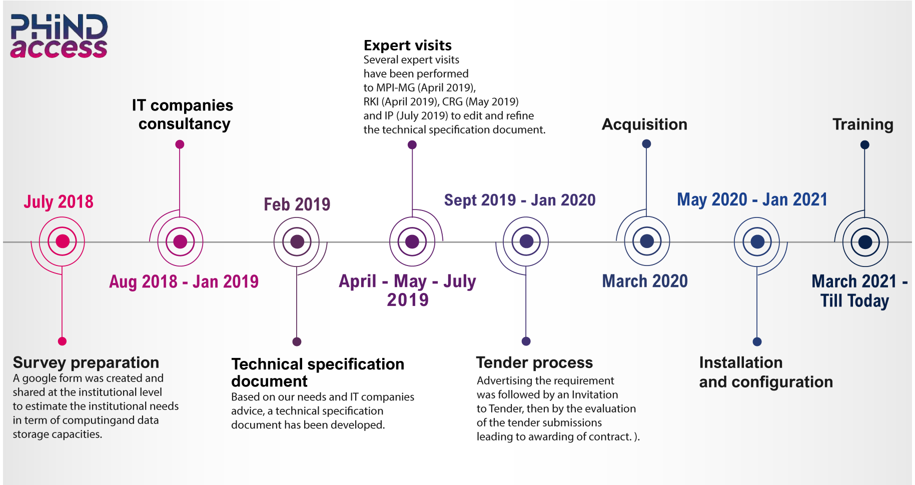

Figure 1. Steps and timeline followed for the acquisition of the infrastructure

case for our institute where a number of bioinformatics analysis including exome analysis, RNA clustering, and RNA-seq data analysis were performed thanks to this infrastructure [8, 9]. Here, we discuss the implementation of an HPC infrastructure at the Institut Pasteur de Tunis (IPT), aiming to enhance omics data management and analysis .

Infrastructure implementation and Timeline

In March 2020, IPT acquired hardware in the frame of the twinning H2020 PHINDaccess project, thanks to the support and funding from the Tunisian Ministry of Scientific Research and Higher Education (MESRS). By January 2021, we introduced "*OMICS*" the implemented HPC facility, envisioning it as a cornerstone institutional bioinformatics platform. This initiative aimed to significantly enhance IPT users' proficiency in omics data analysis and management (Figure 1).

HPC configuration

To identify the optimal cluster configuration, we conducted a benchmarking study, summarized in Table 1. Simplifying complexity, we implemented NVIDIA Bright Cluster Manager v9.2 to automate setup and management of our Linux Ubuntu 20.04.6 LTS HPC cluster. Leveraging the Easy8 Bright Cluster free version, the system allows swift package installation, updates, and dependency management for various analysis types (Figure 2). Over 60 users access the IPT's HPC infrastructure, supported by a local Glpi Help Desk. SLURM manages job scheduling and resource allocation (Figure 3), while data integrity is maintained through nightly automated backups and weekly snapshots, ensuring reliable system recovery. Besides the increasing number of users, till today, we have successfully connected our HPC cluster to multiple sequencing platforms using the SMB protocol, enabling efficient data storage and transfer. This setup supports the management of approximately 5 TB of data generated weekly. This integration ensures a streamlined workflow and enhances our data processing capabilities.

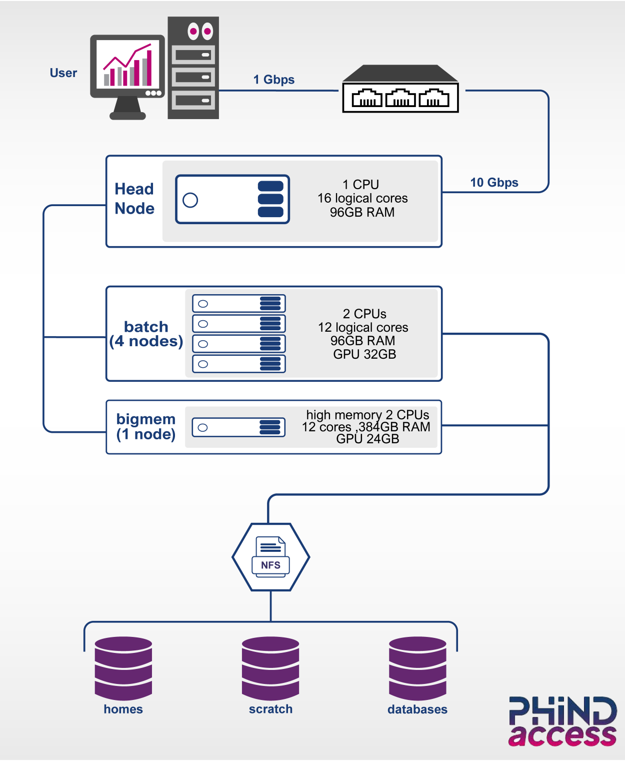

Figure 2. Diagram of the OMIX architecture
"*OMICS*" cluster includes one head/login node, five compute nodes including a bigmem (high memory) node. All compute nodes are equipped with GPUs. "*OMICS*" consists of 272CPU cores, 128GPU cores, 760GB memory, and 95TB storage. Software packages and bioinformatics tools can be installed via Conda or through modules that are either pre-installed or can be installed upon request from the system administrator. Configuration includes a single partition for job submission, and the interconnection between login and compute nodes takes place via a 10Gbps switch data center, featuring high throughput and low latency. "*OMICS*" achieves a peak performance of 9.0 Tflops according to (LinPack benchmarks) [10].

Table 1. Benchmarking of some cluster management tools based on some selected features

Features	Bright Cluster Manager	OpenHPC	Aspen Systems Cluster Management	ClusterVisor
Installation	Offers a simplified installation.	Requires manual installation	Aspen provides command line tools on the clusters for imaging, remote power and sensor programs	Offers a simple installation and easy accession
Configuration	Configuration process may include automated provisioning and updates.	Configuration of individual HPC software components may require a high degree of expertise.	Possibility of streamlining and configuring diverse management utilities while also handling the transfer of users previous utilities licenses.	Use of a dashboard for an easy configuration process via the command lines and a graphical interface.
Support	Typically comes with commercial support options.	Community-driven with community support	Compatible with most Linux distributions and is supported for the life of the cluster	Disponibility of a support team
Documentation	Extensive documentation	Documentation may vary in completeness		Availability of a detailed manual
Cost	Usually involves licensing fees and support costs	OpenHpc is open-source and free to use, but costs may be incurred for hardware, support and additional software components	Some resource manager and scheduler combinations are open source with no charges, while some are commercial products that need to be purchased.	Involves licensing fees and possibly support costs
Cluster size	Any size	Any size	Few to large groups of nodes.	Any size
Monitoring	All aspects involving the cluster's usage and stats can be easily monitored via the integrated dashboard	All cluster stats can be monitored via ganglia, a scalable monitoring system for HPC.	All aspects of the cluster can be monitored, including performance/utilization, network saturation, power consumption, temperature monitoring.	All aspects involving the cluster's usage and stats can be easily monitored via the integrated dashboard

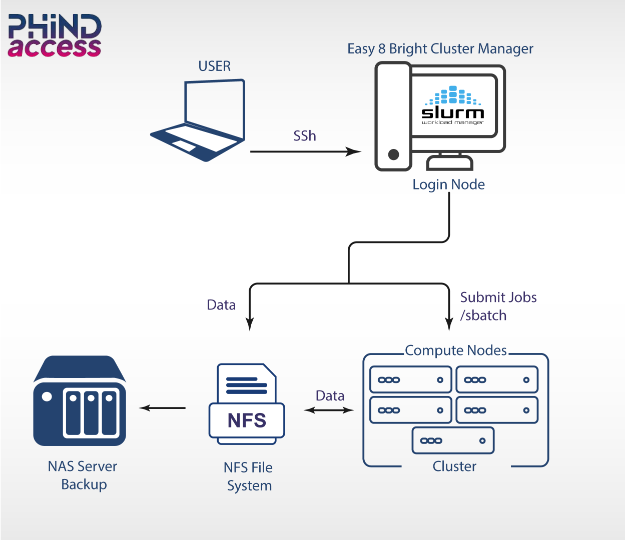

Figure 3. HPC cluster components and architecture
Users have to connect to the "*OMICS*" cluster head/login node via ssh linux command. The Simple Linux Utility for Resource Management (SLURM) (https://slurm.schedmd.com/documentation.html) open-source cluster management system orchestrates the job scheduling and allocates resources. Jobs can be submitted and deployed on compute nodes through running bash scripting using the "sbatch" command or using the slurm interactive mode via the "srun" command.

Trainings delivery

Training has been an integral component of the HPC facility mindset since its launch and enables knowledge sharing across MSc, PhD students and researchers within IPT. The objective is to improve and diversify the educational component of the HPC system by providing courses on a more regular basis and targeting a wider audience (Figure 4).

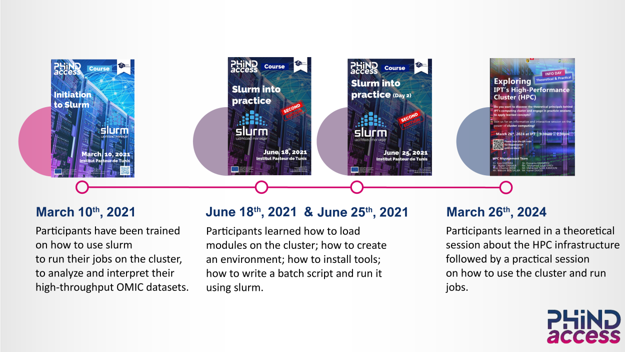

Figure 4. Training activities
An introductory course named "Initiation to Slurm" followed by another course "Slurm into practice" have been organized, aimed at familiarizing IPT users with the infrastructure, with bash scripting and job submission in SLURM using the "sbatch" and "srun"commands.

Challenges

Limited skilled workforce

The administration of Linux HPC systems demands highly skilled SysAdmins to manage the infrastructure efficiently. However, structured training programs for HPC SysAdmins are scarce and typically limited to university-level courses tailored for postgraduate computer scientists. While public resources exist for HPC user training, SysAdmins often learn through hands-on experience, which can burden HPC resources and result in inefficient management. Given the diverse institutional needs and infrastructures, a standardized training for SysAdmins is unlikely. Additionally, the lack of IT professionals skilled in Linux OS and omics data management further complicates the issue, disrupting workflows that require expertise in Linux, command lines, and Shell/Bash scripting. Windows administrators may need to acquire Linux tools, and SysAdmins

should focus on specialized tasks such as OS maintenance, user management, and data security, rather than handling all technical assistance aspects.

Lack of onsite HPC network experts

The local IT companies lacked experience in managing this kind of infrastructure in the context of omics data analysis, thus forcing the SysAdmin to initiate the installation and configuration from scratch, which resulted in major issues. Therefore, prior familiarity with HPC architecture is crucial for SysAdmins.

Recommendations for HPC Implementation

The challenges faced led to the development of a priority recommendations list for teams wishing to implement similar infrastructures.

Technical recommendations

Configuring the HPC cluster is crucial to ensure its efficiency, stability, and security. Numerous software options exist; however, the best solution that we suggest, especially for smaller clusters (less than 8 nodes), is the Easy8 Bright Cluster Manager Solution due to its comprehensive management capabilities and free certification. Installing bioinformatics tools on an HPC cluster also poses challenges for both users and SysAdmins, particularly when different software versions are required. Thus based on our experience, the "module" system, (common on supercomputers), streamlines software usage, while Conda/Anaconda/Miniconda allows users to create Python environments and install the necessary tools efficiently.

User training and support activities

Based on our experience, prioritizing user training in our HPC facility is a major milestone by offering comprehensive courses in SLURM and bash scripting, as this is essential for efficient job management and resource allocation (detailed in Figure 4). Establishing detailed guidelines, SOPs, and user policies is crucial for proper system use and maintenance, enhancing system efficiency and empowering researchers to actively contribute to its development and sustainability. Additionally, implementing a Google form for access requests ensures that users will demonstrate adequate knowledge of command line, SLURM, and bash scripting, further solidifying the commitment to a well-informed and capable user base.

HPC Sustainability

HPC sustainability involves ensuring the long-term viability of infrastructures based on various metrics and criteria. It encompasses human resources, infrastructure hardware and software optimizations. Key factors include investing in specialized human resources, providing long-term contracts or permanent employment to maintain stability. Adequate funding is essential for the continuous expansion and enhancement of the infrastructure. Additionally, creating a cluster management committee is vital to knowledge sharing and documentation, keeping up with SysAdmin duties and fostering collaboration with other facilities managing HPCs in the LMIC countries, ensuring that the infrastructure remains efficient and effective.

Conclusion

The IPT HPC infrastructure journey, from challenges to deployment, offers insights for LMICs. Key recommendations cover cluster configuration, management, sustainability, and user training, forming an efficient implementation framework. Such initiatives promote equitable access to computing resources, fostering global scientific collaboration and advancing scientific development.

Data Availability

Not applicable

Conflicts of Interest

The authors declare that there is no conflict of interest regarding the publication of this paper.

List of abbreviations

HPC: High Performance Computing; IPT: Institut Pasteur de Tunis; LMICs: Low and middle income countries; MESRS: Tunisian Ministry of Scientific Research and Higher Education; SLURM: Simple Linux Utility for Resource Management; SOPs: Standard Operating Procedures; SMB: Server Message Block; OS: Operating System; SysAdmin: System Administrator

Ethical Approval (optional)

'Not applicable'.

Consent for publication

'Not applicable'

Competing Interests

'The author(s) declare that they have no competing interests'.

Funding

This work was supported by the Tunisian Ministry of Higher Education and Scientific Research and the Institut Pasteur de Tunis. The PHINDaccess bioinformatics infrastructure has been funded by the Tunisian Ministry of Higher Education and Scientific Research. The study was supported by the European project PHINDaccess: Strengthening Omics data analysis capacities in pathogen-host interaction (Grant agreement ID: 811034).

Author's Contributions

Kais Ghedira: Designed and supervised the study, wrote the manuscript, reviewed the manuscript
Oussema Khamessi, Chaima Hkimi, Selim Kamoun: reviewed the manuscript, worked on the figures generation
Nader Dhamer, Wahbi Belhadj, Youssef Ghorbal: Implemented and configured the HPC cluster, wrote the manuscript
Youssef Ghorbal: Supervised the study, Implemented and configured the HPC cluster, wrote the manuscript
Kamel Daassi, Wassim Ben Salah, Houcemeddine Othman: contributed in the writing of the manuscript

Acknowledgements

We would like to acknowledge Pr Samia Menif, General Director of the Institut Pasteur de Tunis as well as Pr Helmi Mardassi, the Principal Investigator of the PHINDaccess project and the Tunisian Ministry of higher education and scientific research (MESRS) for funding the acquisition of the bioinformatics infrastructure. We also acknowledge the help of the HPC Core Facility of the Institut Pasteur for this work.

References

1. Zhang Y, Cheng Y, Jia K, Zhang A. Opportunities for computational techniques for multi-omics integrated personalized medicine. Tsinghua Science and Technology 2014;19(6):545–558.
2. Yu XT, Zeng T. Integrative analysis of omics big data. Computational Systems Biology: Methods and Protocols 2018;p. 109–135.
3. Tulasi B BS Rupali Sunil Wagh. High Performance Computing and Big Data Analytics - Paradigms and Challenges. International Journal of Computer Applications 2015 April;116(2):28–33. https://ijcaonline.org/archives/volume116/number2/20311-2356/.
4. Leff D, Yang GZ. Big Data for Precision Medicine. Engineering 2015 09;1:277.
5. UNESCO. UNESCO Recommendation on Open Science 2021;https://unesdoc.unesco.org/ark:/48223/pf0000379949.
6. Courneya JP, Mayo A. High-performance computing service for bioinformatics and data science. Journal of the Medical Library Association: JMLA 2018;106(4):494.
7. Kurc T, Hastings S, Kumar V, Langella S, Sharma A, Pan T, et al. HPC and Grid Computing for Integrative Biomedical Research. The international journal of high performance computing applications 2009 08;23:252.
8. Hkimi C, Kamoun S, Khamessi O, Ghedira K. Mycobacterium tuberculosis-THP-1 like macrophages protein-protein interaction map revealed through dual RNA-seq analysis and a computational approach. Journal of Medical Microbiology 2024;73(2):001803.
9. Ghedira K, Dallali H, Ardhaoui M, Bouslema Z, Hamdi Y, Feki Ben-Salah S, et al. PHINDaccess Hackathons for COVID-19 and Host-Pathogen Interaction: Lessons Learned and Recommendations for Low-and Middle-Income Countries. BioMed Research International 2023;2023.
10. Dongarra J, Luszczek P, Petitet A. The LINPACK Benchmark: past, present and future. Concurrency and Computation: Practice and Experience 2003 08;15:803–820.

Figure

Click here to access/download;Figure;Fig1.tiff

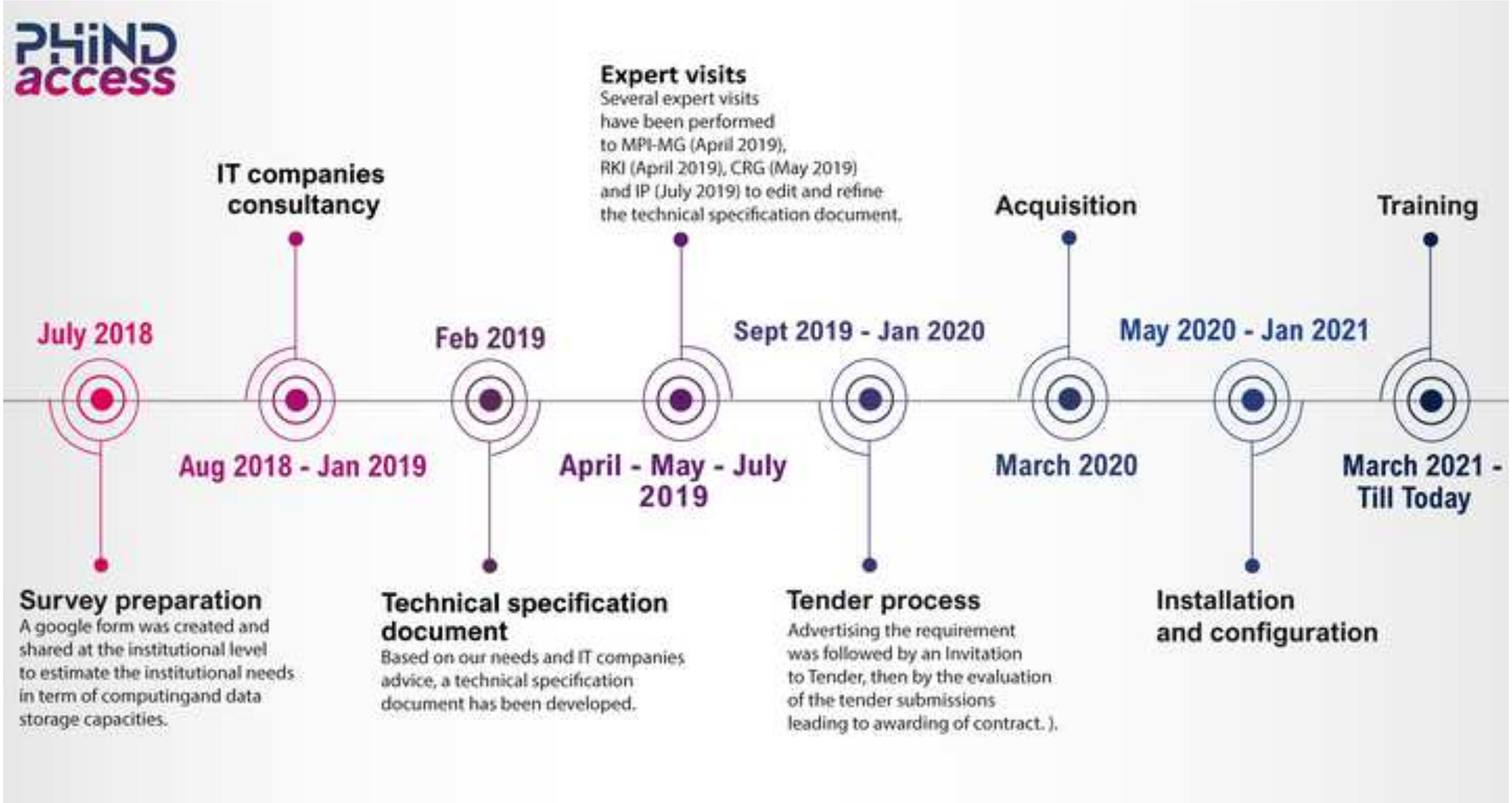

Figure

Click here to access/download;Figure;Figure 2.tif

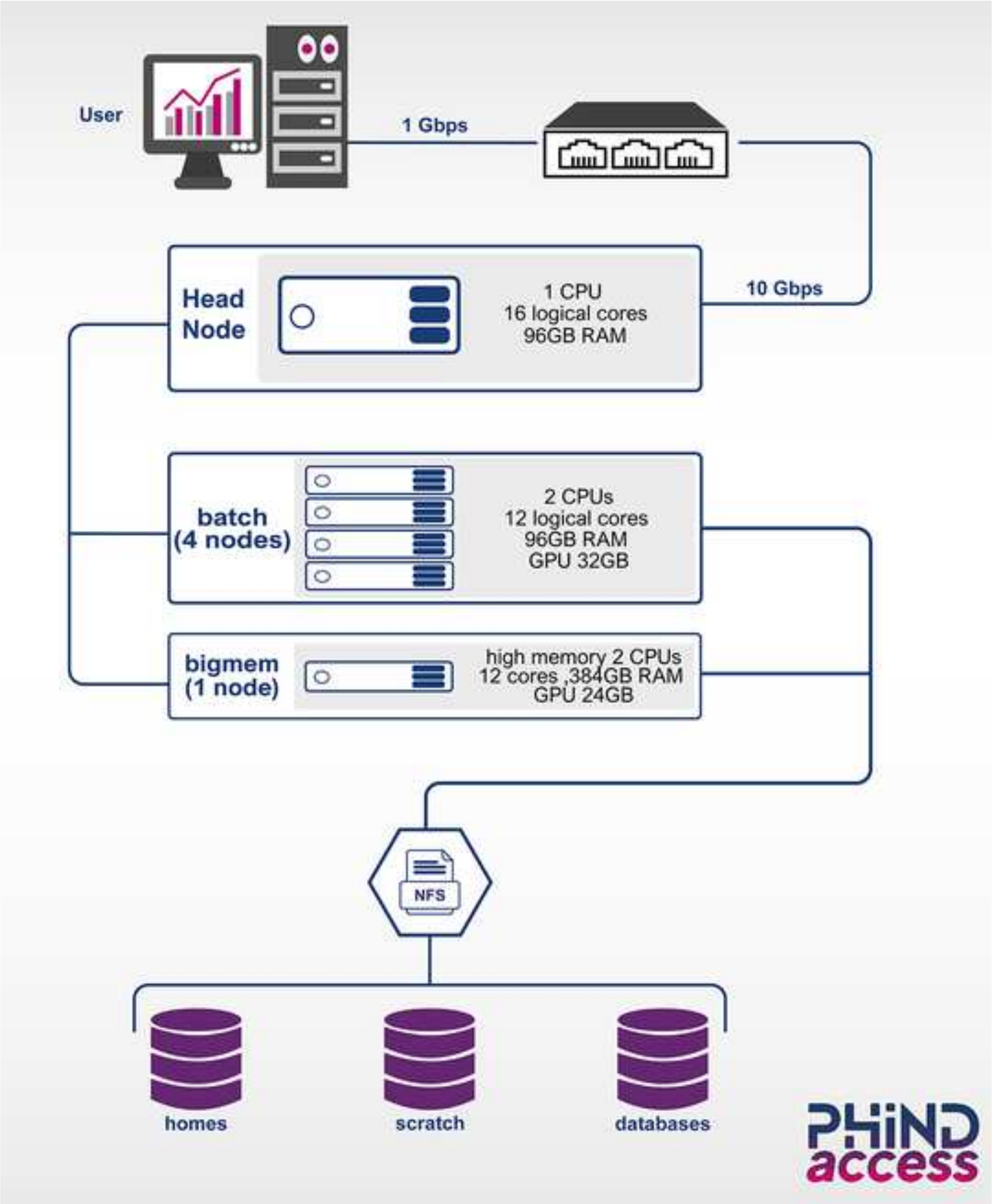

Click here to access/download;Figure;Figure 3.tif

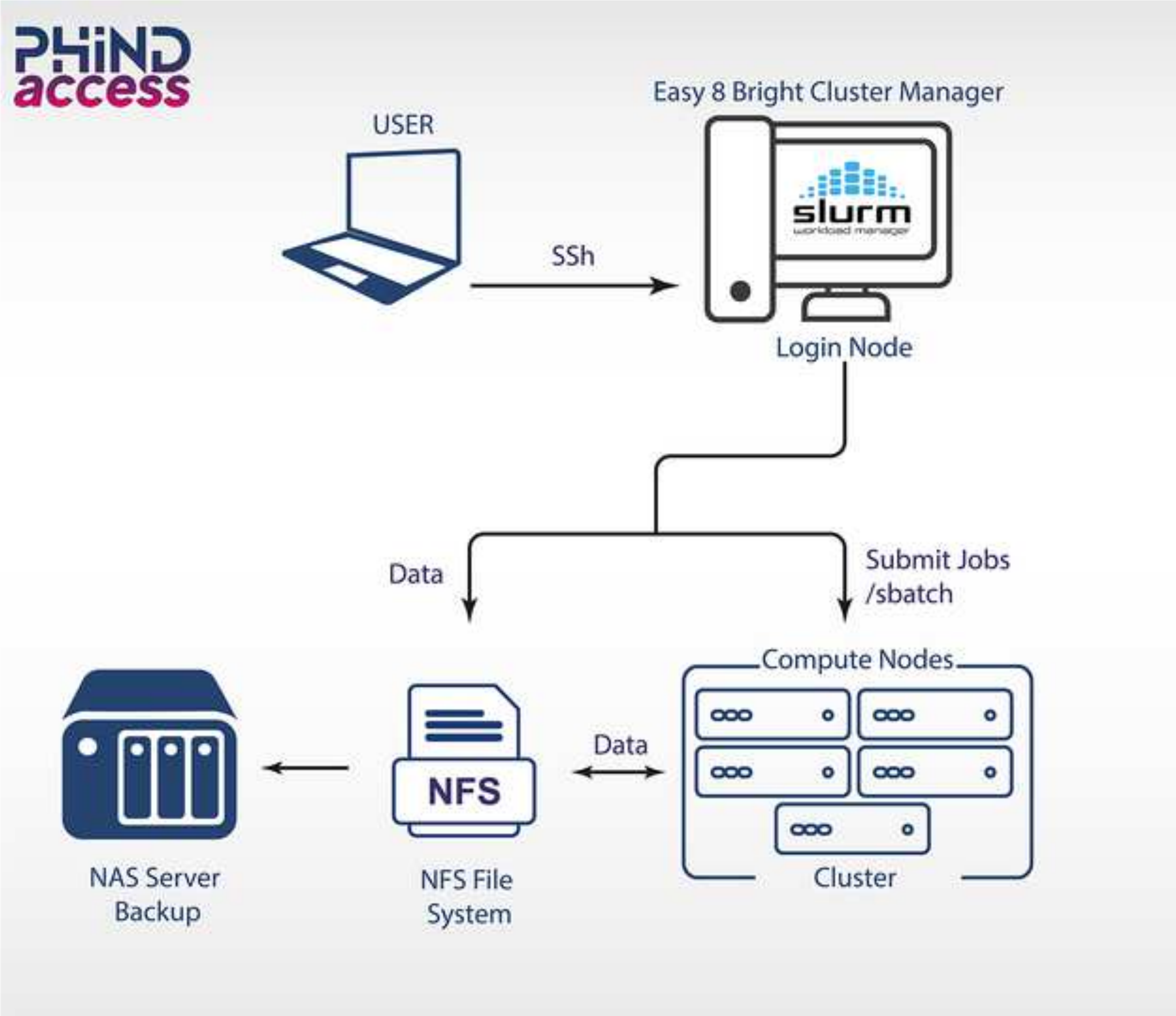

Figure
Click here to access/download;Figure;Fig4.tiff

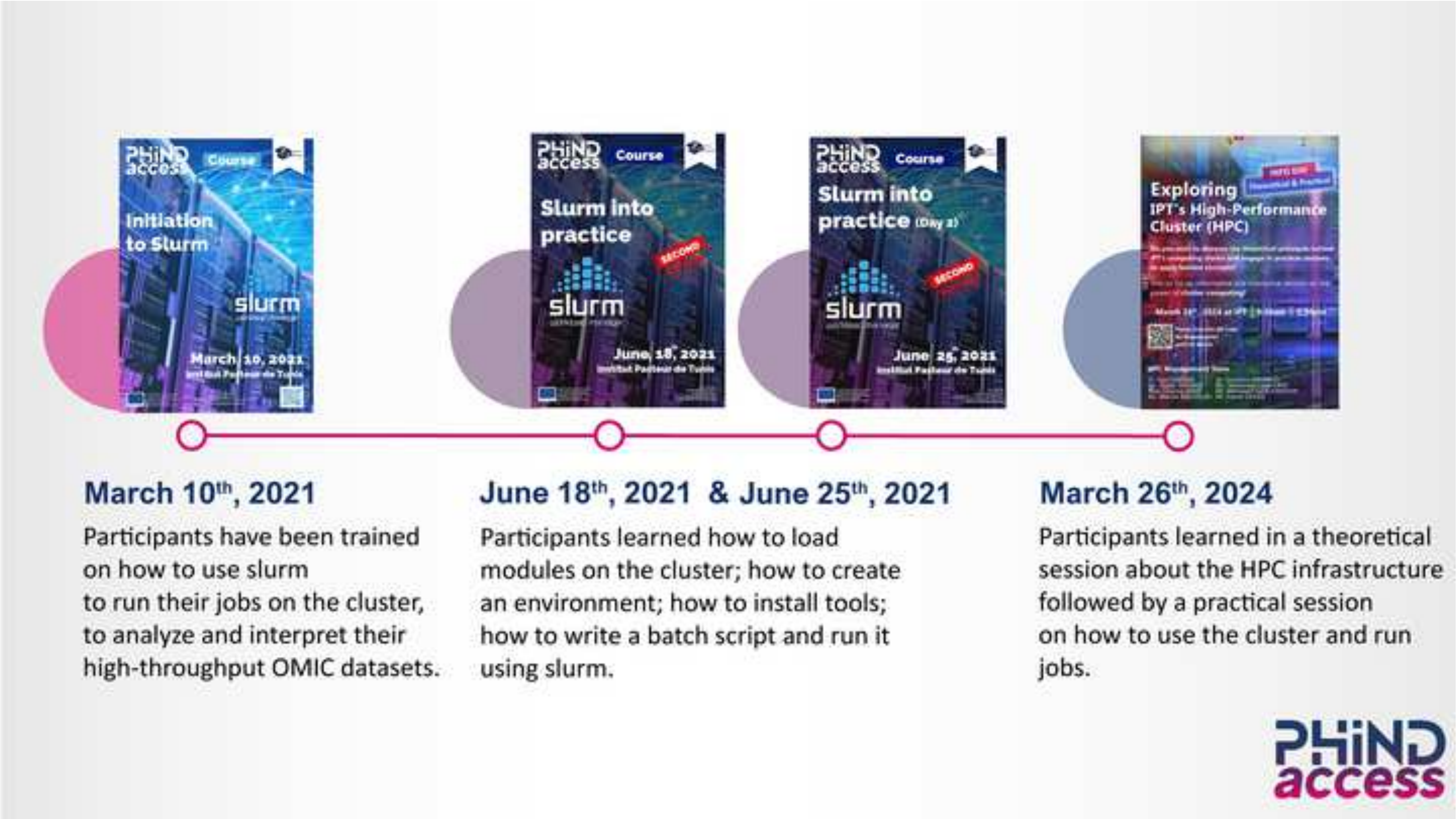

Personal Cover Click here to access/download;Personal Cover;cover_letter_response2reviewers.docx

Kais Ghedira
13, Place Pasteur B.P.74
Tunis, 1002, Tunisia.
+216 53 370 404
kais.ghedira@pasteur.tn

Dear Editors,

We are pleased to address this letter to thank you and thank the reviewers for your valuable comments aiming to improve the quality of the manuscript with the accession number Manuscript GIGA-D-24-00092, submitted by Kais Ghedira et al in your highly esteemed *GigaScience* journal as a commentary article, to make it more clear and understandable for the journal readers.

Please find enclosed the revised version of the manuscript with the title: "**Design and Implementation of a scalable High Performance Computing (HPC) Cluster for OMICS data analysis: Achievements, challenges and recommendations in LMICs**" by Ghedira and collaborators. As you already know the paper outlines the steps undertaken in order to implement an operational HPC cluster at the Institut Pasteur de Tunis, Tunisia. We also highlight the difficulties and challenges faced during the whole process as well as recommendations that may prove to be useful to other organizations and institutions in LMICs that are keen to set up local HPC facilities to help enhance bioinformatics analysis.

All the comments raised by the reviewers and the editor have been carefully addressed. Point by point responses can be found as an annexe to this cover letter. We have now better formulate our ideas and take into account the reviewers' concerns when revising the manuscript. We hope that the reviewers will find our answers and edits more convincing. We hope that this manuscript will be now suitable for publication in the GigaScience journal.

We believe that our work here is eligible for publication in *GigaScience*. Indeed, on premise HPC (high-performance computing) clusters are essential today in bioinformatics, multi OMICS data analysis, IA and the handling and processing of big data generated from Omics high-throughput sequencing technologies. Regarding the funds, the presented implementation of the HPC bioinformatics infrastructure was supported by the Tunisian Ministry of Higher Education and Scientific Research and the Institut Pasteur de Tunis. The study was also supported by the European H2020 project entitled PHINDaccess: Strengthening Omics data analysis capacities in pathogen-host interaction (Grant agreement ID: 811034).

This manuscript has not been published and is not under consideration for publication elsewhere. We have no conflicts of interest to disclose.

Thank you for your consideration!
Sincerely,

The corresponding author; Dr. Kais Ghedira, Institut Pasteur de Tunis

Laboratory of Bioinformatics, Biomathematics and Biostatistics (LR20IPT09), Pasteur Institute of Tunis, University of Tunis El Manar, Tunis, 1002, Tunisia.
Email: Kais.ghedira@pasteur.tn

Annexe

Response to the editor and the reviewers

Having had a look at the reviewer's suggestions, we feel that it is too much for a Commentary - the reviewer would like more information added, but we suggest to keep it to our shorter Commentary format.

However, the following revisions must be made:

- Please update the writing to read less like a tutorial and to read more of the lessons learned - this is what we did, this is how we did it, and this is what we've learnt. Presenting it in this way will be more useful for the greater community.

-We thank the editor for this important comment. We took it into account and tried to update the writing style within the text in order to appear more like learned lessons and less like a tutorial as initially proposed.

- Please update the figure descriptions as per the Reviewer's comments and also Table 1.

Try and include as much information as possible without having to add more references (as we have a strict Reference maximum of 10 for Commentaries).

-We appreciate the editor's suggestion. The figure descriptions have been updated as well as table1 in accordance with the suggestions. References have also been kept at a maximum of 10.

Reviewer reports:

Reviewer #1: Design and Implementation of a scalable High Performance Computing (HPC) Cluster for OMICS data analysis: Achievements, challenges and recommendations in LMICs

The paper introduces a comprehensive exploration of the design and implementation of an HPC cluster tailored for OMICS data analysis, particularly focusing on its application within Low- and Middle-Income Countries (LMICs). This topic holds relevance for both the HPC and bioinformatics communities. While the paper delves into specialized HPC technologies crucial for real-world bioinformatics studies, it lacks clarity in its **objectives and could better address the aspects of**

achievements, challenges, and recommendations specific to LMICs. Additionally, the organization of paragraphs requires refinement, as the current presentation leans towards a tutorial-style description rather **than a concise scientific review or commentary**.

-We thank the reviewer for this valuable comment. The objectives and the organization of paragraphs has been addressed and updated according to the reviewer comments.

The authors' objective is to elucidate the insights and hurdles encountered during the deployment of a cluster dedicated to OMICS data analysis, aiming to provide valuable guidance for LMICs grappling with similar challenges. Although the paper briefly touches on related topics such as artificial intelligence, big data, data science, and open science infrastructure, a clearer integration of these elements into the HPC environment is warranted. Notably, Figure 1 outlines the timeline of infrastructure acquisition spanning from 2018 to 2021; however, considering the elapsed time since then (now April 2024), **potential updates or changes to the environment should be acknowledged**. Furthermore, **incorporating experiences from other LMIC HPC centers into the discussion could enrich the paper**. It is recommended to update references to include more recent and pertinent sources.

-We thank the reviewer for this comment. Figure 1 has been updated and details regarding the updates related to the environment have been added in HPC configuration section : "Till today, we have successfully connected our HPC cluster to multiple sequencing platforms using the SMB protocol, enabling efficient data storage and transfer. This setup supports the management of approximately 5 TB of data generated weekly. This integration ensures a streamlined workflow and enhances our data processing capabilities."

Regarding the following point addressing the importance of incorporating other LMIC HPC centers experience into the discussion, as to our knowledge, there isn't detailed and specific works treating the design and implementation of such infrastructures within research facilities in LMIC countries. This makes it hard to incorporate such aspects within this work.

Moreover, enhancing the clarity of experimental and comparative benchmarks mentioned in Table 1 would be beneficial. Additionally, the scarcity of human resources proficient in Linux and OMICS data analysis is highlighted, underscoring the interplay between computer science and bioinformatics in maintaining OMICS infrastructure. **Figures 2, 3, and 4 require more detailed descriptions within the**

text, elucidating their significance, particularly regarding the relevance of Figure 4 to the paper's objectives.

-We thank the reviewer for this important suggestion. Table 1 has been reviewed in that regard. The suggested additions regarding the figures 2, 3, and 4 have been taken into account. However, in order to also take into account the editor's comment who suggested to not extend more the text so it would still fit the commentary format, the details suggested by the reviewer have been added into the figures description section rather than within the text. This enables us to make the reviewers suggested adjustments while still taking into account the editor's comments.

Recommendations for HPC Implementation:

To augment the paper's impact, the authors should provide a concrete and specific description of a bioinformatics case study, moving beyond the current generalized approach. The current presentation **resembles a manual or tutorial, lacking the depth** required for a scientific discourse. **This adjustment would elevate the paper's value by offering practical insights derived from real-world applications.**

-We appreciate the reviewer's suggestion. We added 2 examples in that regard. The first is one of our recent studies involving the prediction of protein-protein interactions through dual RNA-seq analysis in the context of *Mycobacterium tuberculosis* infection (reference 8 in the manuscript) that used this infrastructure. The second one is a hackathon organized in the frame of the twinning project PHINDaccess aiming to develop portable and reproducible genomics analysis pipelines for SARS-CoV-2 and that was made possible thanks to this infrastructure.
