# Supplementary material for: Design and implementation of a scalable high-performance computing (HPC) cluster for omics data analysis: achievements, challenges and recommendations in LMICs
Source: Gigascience. 2024 Aug 22;13:giae060. doi: 10.1093/gigascience/giae060 (PMC11340639; doi:10.1093/gigascience/giae060)
Supplement: giae060_Response_to_Reviewer_Comments_Original_Submission [file giae060_response_to_reviewer_comments_original_submission.pdf]

Author's Response To Reviewer Comments

Response to the editor and the reviewers

Having had a look at the reviewer's suggestions, we feel that it is too much for a Commentary - the reviewer would like more information added, but we suggest to keep it to our shorter Commentary format.

However, the following revisions must be made:

- Please update the writing to read less like a tutorial and to read more of the lessons learned - this is what we did, this is how we did it, and this is what we've learnt. Presenting it in this way will be more useful for the greater community.
-We thank the editor for this important comment. We took it into account and tried to update the writing style within the text in order to appear more like learned lessons and less like a tutorial as initially proposed.

- Please update the figure descriptions as per the Reviewer's comments and also Table 1.
Try and include as much information as possible without having to add more references (as we have a strict Reference maximum of 10 for Commentaries).
-We appreciate the editor's suggestion. The figure descriptions have been updated as well as table1 in accordance with the suggestions. References have also been kept at a maximum of 10.

Reviewer reports:

Reviewer #1: Design and Implementation of a scalable High Performance Computing (HPC) Cluster for OMICS data analysis: Achievements, challenges and recommendations in LMICs

The paper introduces a comprehensive exploration of the design and implementation of an HPC cluster tailored for OMICS data analysis, particularly focusing on its application within Low- and Middle-Income Countries (LMICs). This topic holds relevance for both the HPC and bioinformatics communities. While the paper delves into specialized HPC technologies crucial for real-world bioinformatics studies, it lacks clarity in its objectives and could better address the aspects of achievements, challenges, and recommendations specific to LMICs. Additionally, the organization of paragraphs requires refinement, as the current presentation leans towards a tutorial-style description rather than a concise scientific review or commentary.
-We thank the reviewer for this valuable comment. The objectives and the organization of paragraphs has been addressed and updated according to the reviewer comments.

The authors' objective is to elucidate the insights and hurdles encountered during the deployment of a cluster dedicated to OMICS data analysis, aiming to provide valuable guidance for LMICs grappling with similar challenges. Although the paper briefly touches on related topics such as artificial intelligence, big data, data science, and open science infrastructure, a clearer integration of these elements into the HPC environment is warranted. Notably, Figure 1 outlines the timeline of infrastructure acquisition spanning from 2018 to 2021; however, considering the elapsed time since then (now April 2024), potential updates or changes to the environment should be acknowledged. Furthermore, incorporating experiences from other LMIC HPC centers into the discussion could enrich the paper. It is recommended to update references to include more recent and pertinent sources.
-We thank the reviewer for this comment. Figure 1 has been updated and details regarding the updates related to the environment have been added in HPC configuration section : "Till today, we have successfully connected our HPC cluster to multiple sequencing platforms using the SMB protocol, enabling efficient data storage and transfer. This setup supports the management of approximately 5 TB of data generated weekly. This integration ensures a streamlined workflow and enhances our data processing capabilities."
Regarding the following point addressing the importance of incorporating other LMIC HPC centers

experience into the discussion, as to our knowledge, there isn’t detailed and specific works treating the design and implementation of such infrastructures within research facilities in LMIC countries. This makes it hard to incorporate such aspects within this work.

Moreover, enhancing the clarity of experimental and comparative benchmarks mentioned in Table 1 would be beneficial. Additionally, the scarcity of human resources proficient in Linux and OMICS data analysis is highlighted, underscoring the interplay between computer science and bioinformatics in maintaining OMICS infrastructure. Figures 2, 3, and 4 require more detailed descriptions within the text, elucidating their significance, particularly regarding the relevance of Figure 4 to the paper's objectives.
-We thank the reviewer for this important suggestion. Table 1 has been reviewed in that regard. The suggested additions regarding the figures 2, 3, and 4 have been taken into account. However, in order to also take into account the editor’s comment who suggested to not extend more the text so it would still fit the commentary format, the details suggested by the reviewer have been added into the figures description section rather than within the text. This enables us to make the reviewers suggested adjustments while still taking into account the editor’s comments.

Recommendations for HPC Implementation:
To augment the paper's impact, the authors should provide a concrete and specific description of a bioinformatics case study, moving beyond the current generalized approach. The current presentation resembles a manual or tutorial, lacking the depth required for a scientific discourse. This adjustment would elevate the paper's value by offering practical insights derived from real-world applications.
-We appreciate the reviewer's suggestion. We added 2 examples in that regard. The first is one of our recent studies involving the prediction of protein-protein interactions through dual RNA-seq analysis in the context of Mycobacterium tuberculosis infection (reference 8 in the manuscript) that used this infrastructure. The second one is a hackathon organized in the frame of the twinning project PHINDaccess aiming to develop portable and reproducible genomics analysis pipelines for SARS-CoV-2 and that was made possible thanks to this infrastructure.
