# Supplementary material for: Design and implementation of a scalable high-performance computing (HPC) cluster for omics data analysis: achievements, challenges and recommendations in LMICs
Source: Gigascience. 2024 Aug 22;13:giae060. doi: 10.1093/gigascience/giae060 (PMC11340639; doi:10.1093/gigascience/giae060)
Supplement: giae060_Reviewer_1_Report_Original_Submission — Kary Ocana -- 4/29/2024 Reviewed [file giae060_reviewer_1_report_original_submission.pdf]

Reviewer Report

Title: Design and Implementation of a scalable High Performance Computing (HPC) Cluster for OMICS data analysis: Achievements, challenges and recommendations in LMICs

Version: Original Submission **Date:** 4/29/2024

Reviewer name: Kary Ocana

Reviewer Comments to Author:

Design and Implementation of a scalable High Performance Computing (HPC) Cluster for OMICS data analysis: Achievements, challenges and recommendations in LMICsThe paper introduces a comprehensive exploration of the design and implementation of an HPC cluster tailored for OMICS data analysis, particularly focusing on its application within Low- and Middle-Income Countries (LMICs). This topic holds relevance for both the HPC and bioinformatics communities. While the paper delves into specialized HPC technologies crucial for real-world bioinformatics studies, it lacks clarity in its objectives and could better address the aspects of achievements, challenges, and recommendations specific to LMICs. Additionally, the organization of paragraphs requires refinement, as the current presentation leans towards a tutorial-style description rather than a concise scientific review or commentary.The authors' objective is to elucidate the insights and hurdles encountered during the deployment of a cluster dedicated to OMICS data analysis, aiming to provide valuable guidance for LMICs grappling with similar challenges. Although the paper briefly touches on related topics such as artificial intelligence, big data, data science, and open science infrastructure, a clearer integration of these elements into the HPC environment is warranted. Notably, Figure 1 outlines the timeline of infrastructure acquisition spanning from 2018 to 2021; however, considering the elapsed time since then (now April 2024), potential updates or changes to the environment should be acknowledged. Furthermore, incorporating experiences from other LMIC HPC centers into the discussion could enrich the paper. It is recommended to update references to include more recent and pertinent sources.Moreover, enhancing the clarity of experimental and comparative benchmarks mentioned in Table 1 would be beneficial. Additionally, the scarcity of human resources proficient in Linux and OMICS data analysis is highlighted, underscoring the interplay between computer science and bioinformatics in maintaining OMICS infrastructure. Figures 2, 3, and 4 require more detailed descriptions within the text, elucidating their significance, particularly regarding the relevance of Figure 4 to the paper's objectives.Recommendations for HPC Implementation:To augment the paper's impact, the authors should provide a concrete and specific description of a bioinformatics case study, moving beyond the current generalized approach. The current presentation resembles a manual or tutorial, lacking the depth required for a scientific discourse. This adjustment would elevate the paper's value by offering practical insights derived from real-world applications..

Level of Interest

Please indicate how interesting you found the manuscript: Choose an item.

Quality of Written English

Please indicate the quality of language in the manuscript: Choose an item.

Declaration of Competing Interests

Please complete a declaration of competing interests, considering the following questions:

- Have you in the past five years received reimbursements, fees, funding, or salary from an organisation that may in any way gain or lose financially from the publication of this manuscript, either now or in the future?
- Do you hold any stocks or shares in an organisation that may in any way gain or lose financially from the publication of this manuscript, either now or in the future?
- Do you hold or are you currently applying for any patents relating to the content of the manuscript?
- Have you received reimbursements, fees, funding, or salary from an organization that holds or has applied for patents relating to the content of the manuscript?
- Do you have any other financial competing interests?
- Do you have any non-financial competing interests in relation to this paper?

If you can answer no to all of the above, write 'I declare that I have no competing interests' below. If your reply is yes to any, please give details below.

I declare that I have no competing interests'

I agree to the open peer review policy of the journal. I understand that my name will be included on my report to the authors and, if the manuscript is accepted for publication, my named report including any attachments I upload will be posted on the website along with the authors' responses. I agree for my report to be made available under an Open Access Creative Commons CC-BY license (http://creativecommons.org/licenses/by/4.0/). I understand that any comments which I do not wish to be included in my named report can be included as confidential comments to the editors, which will not be published.

Choose an item.

To further support our reviewers, we have joined with Publons, where you can gain additional credit to further highlight your hard work (see: https://publons.com/journal/530/gigascience). On publication of this paper, your review will be automatically added to Publons, you can then choose whether or not to claim your Publons credit. I understand this statement.

Yes Choose an item.
